# Combining Network Pharmacology, Molecular Docking, and Integrative Studies to Explore the Mechanism of *Helminthostachys zeylanica* in Alleviating Ulcerative Colitis

**DOI:** 10.1002/fsn3.71139

**Published:** 2025-10-28

**Authors:** Chih‐Ting Lin, Li‐Wei Lin, Li‐Ching Chang, Lung‐Yuan Wu, Fan‐Shiu Tsai

**Affiliations:** ^1^ The School of Chinese Medicine for Post‐Baccalaureate I‐Shou University Kaohsiung City Taiwan; ^2^ Department of Chinese Medicine E‐Da Cancer Hospital Kaohsiung City Taiwan; ^3^ Institute of Traditional Medicine National Yang Ming Chiao Tung University Taipei City Taiwan; ^4^ School of Medicine for International Students, College of Medicine I‐Shou University Taipei City Taiwan; ^5^ Wu Lung‐Yuan Chinese Medicine Clinic Taipei City Taiwan

**Keywords:** *Helminthostachys zeylanica*, network pharmacology, TLR4/NF‐κB signaling pathway, ugonin, ulcerative colitis

## Abstract

*Helminthostachys zeylanica* (L.) hook (HZ), has recently gained attention as a potential herbal supplement for managing ulcerative colitis (UC) through its bioactive compounds. To comprehensively investigate HZ's therapeutic effects and underlying mechanisms on UC, we utilized network pharmacology and in vitro and in vivo analyses. The therapeutic potential of HZ was evaluated using a DSS‐induced mouse model of ulcerative colitis, alongside in vitro cellular studies. A network pharmacology approach was first used to predict the active compounds and molecular targets of HZ. Subsequently, integrated experimental techniques—including ELISA, Western blotting, histological analysis, immunofluorescence, flow cytometry, and molecular docking—were employed to validate and support the predicted mechanisms. Network pharmacology analysis identified 15 active compounds in HZ, contributing to its multi‐target synergistic activity and anti‐inflammatory effects. HZ was found to modulate multiple inflammatory pathways, particularly the Toll‐like receptor 4 (TLR4) and NF‐κB signaling pathways, regulating vital inflammatory mediators such as tumor necrosis factor‐α (TNF‐α), interleukin‐6 (IL‐6), and interleukin‐1β (IL‐1β), emphasizing its therapeutic potential in UC. ELISA, Western blot, and histological analyses confirmed that HZ significantly reduced colon inflammation. Immunofluorescence and flow cytometry analyses also demonstrated that HZ alleviated inflammation by regulating TLR4/NF‐κB and CD3 signaling pathways without involving apoptosis. Ultimately, molecular docking further identified core compounds, including Ugonin M, O, K, and R, which exhibited strong binding affinity to critical proteins in the TLR4/NF‐κB pathway, such as TAK1, IKKβ, and RELA, underscoring their role in HZ's anti‐inflammatory mechanisms. Collectively, these findings provide a solid basis for further investigation into the mechanistic effects and broader clinical potential of HZ as a therapeutic approach for UC.

## Graphical abstract

The experiment verified the mechanism of HZ against ulcerative colitis. Verified through in vivo and in vitro experiments, HZ demonstrated partial inhibition of the TLR4/NF‐κB signaling pathway, reduction in inflammatory factors IL‐6 and TNF, and the ability to suppress T cell differentiation and activation in ulcerative colitis. Through network pharmacology and molecular docking, we revealed HZ's intricate multi‐compound, multi‐target, and multi‐pathway intervention in promoting ulcerative colitis, specifically within Toll‐like receptor, NF‐kappa B, T cell receptor, and TNF signaling pathways.

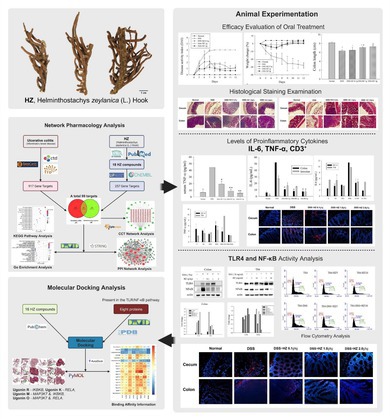

**Abbreviations**
BC: betweenness centrality
CC: closeness centrality
CCT: component‐compound‐target protein
CTD: Comparative Toxicogenomics Database
DAI: Disease Activity Index
DMEM: Dulbecco's modified eagle medium
DSS: dextran sulfate sodium
GO: Gene Ontology
H&E: hematoxylin and eosin
HZ:
*Helminthostachys zeylanica*

IEC: intestinal epithelial cell
IKBKB: Inhibitor of nuclear factor kappa‐B kinase subunit beta
IL‐1β: interleukin‐1 beta
IL‐6: interleukin‐6
IRAK4: interleukin‐1 Receptor‐associated kinase 4
KEGG: Kyoto Encyclopedia of Genes and Genomes
MAP3K7: Mitogen‐activated protein kinase 7
MyD88: myeloid differentiation primary response 88
NADPH: nicotinamide adenine dinucleotide phosphate
NFKBIA: NF‐κB inhibitor alpha
NF‐κB: nuclear factor‐kappa B
PAS: periodic acid‐schiff
PPI: protein–protein interaction
RELA: transcription factor p65
SDS‐PAGE: SDS‐polyacrylamide gel electrophoresis
TAK1: transforming growth factor beta‐activated kinase 1
TBS: Tris‐buffered saline
TCM: traditional Chinese medicine
TLR4: toll‐like receptor 4
TNF‐α: tumor necrosis factor‐alpha
TRAF4: TNF receptor‐associated factor 4
TTD: Therapeutic Target Database
UC: ulcerative colitis

## 1 Introduction

Ulcerative colitis (UC) is an inflammatory bowel disease that begins in the rectum and can extend proximally along the colon, primarily affecting the mucosal and submucosal layers (Arjomand Fard et al. 2023). This condition often leads to impaired intestinal absorption, persistent diarrhea, and weight loss (Kobayashi et al. 2020). The incidence and prevalence of UC in Taiwan have been steadily increasing in recent years (Wei et al. 2021). According to data from the National Health Insurance Research Database, between 2013 and 2022, the incidence rose from 0.54 to 0.95 per 100,000 person‐years, while the prevalence increased from 2.1 to 12.8 per 100,000 people (Kuo et al. 2024; Yen et al. 2019). A 2023 study reported a UC prevalence of approximately 12.4 per 100,000, indicating a growing disease burden that may still be underestimated (Yen et al. 2024). Male patients constitute a higher proportion of UC cases in Taiwan, with a male‐to‐female ratio of about 1.62:1, differing from Western countries, possibly due to smoking and dietary factors (Chen et al. 2022; Hsieh et al. 2022). UC pathogenesis is driven by overproduction of TNF‐α, IL‐1β, and IL‐6, key cytokines that intensify inflammation and gut tissue damage (Nakase et al. 2022; Shahini and Shahini 2023). Th2‐type immune response plays a crucial role in UC, characterized by elevated IL‐1β levels and CD3 activation. This response significantly impairs the colonic epithelial barrier, indicating a stronger Th2 involvement in UC compared to Crohn's disease (Sandborn et al. 2023; Mitsialis et al. 2020). For UC patients who do not respond to corticosteroids, current treatments focus on targeted molecular therapies such as anti‐TNF‐α agents, α4β7 integrin antibodies, IL‐12/23 p40 antibodies, and Janus kinase inhibitors (Elhag et al. 2022; Liang et al. 2024; Liu, Di, et al. 2023; Sinha and Roy 2024). However, 30% to 55% of patients fail to respond during initial treatment, highlighting a significant unmet need for additional therapeutic options (Papamichael et al. 2022; Revés et al. 2021). This gap underscores the potential role of alternative approaches, including traditional herbal medicines and functional foods, as complementary strategies to enhance UC management.

*Helminthostachys zeylanica* (HZ), commonly known as 倒地蜈蚣 (dǎo dì wú gōng), is a traditional Chinese medicinal rhizome from the Ophioglossaceae family, widely found in Southeast Asia, South Asia, and northern Taiwan. Initially recognized for its clinical use in detoxification, pain relief, and UC treatment, HZ's rich bioactive profile is now gaining attention in food science as a potential functional ingredient, supporting its development as a nutraceutical or functional food for complementary therapy. The plant's key bioactive compounds include flavonoids and plant sterols, with quercetin and ugonins being the most prominent. Beyond traditional uses, HZ demonstrates significant therapeutic effects in models of neuroinflammation, osteoporosis, acute lung injury, asthma, and UC, primarily through modulation of inflammatory mediators and enhancement of antioxidant defenses (Wu, Kao, et al. 2017; Liou et al. 2017; Huang et al. 2017; Lin et al. 2023). Ethanol and water extracts of HZ effectively inhibit inflammatory responses by reducing proinflammatory cytokines such as TNF‐α and IL‐6, thereby improving tissue damage (Liou et al. 2017; Huang et al. 2017). The primary bioactive compounds of HZ are flavonoids and plant sterols, notably quercetin and the ugonin family (Wu et al. 2014; Yang et al. 2016). Among them, Ugonin L exhibits potent anti‐inflammatory activity by regulating MAPK and NF‐κB signaling pathways, suppressing osteoclast differentiation, and promoting osteoclast apoptosis via inhibition of RANKL‐induced signaling, thus mitigating bone loss (Liu, Ho, et al. 2023; Huang et al. 2020). Ugonin K shows selective cytotoxicity against skin cancer cells by inducing cell cycle arrest and apoptosis, while exerting antioxidant effects that reduce oxidative stress and protect normal cells (Chan et al. 2013). Additionally, Ugonin M contributes to the anti‐inflammatory effect by inhibiting TLR4‐mediated MAPK and NF‐κB pathways and enhancing antioxidant enzyme activities (Wu, Huang, et al. 2017; Wu et al. 2018). Collectively, the ugonin family exhibits diverse bioactivities including anti‐inflammatory, antioxidant, anti‐osteoporotic, and anti‐cancer effects, making them key components responsible for HZ's multifaceted benefits.

Network pharmacology, widely applied in natural product and food science research, integrates bioinformatics, systems biology, and pharmacology to systematically reveal drug mechanisms and predict bioactive compound effects, thereby guiding experimental design and nutraceutical development (Meimei et al. 2022; Long et al. 2022). In our study, we used network pharmacology to analyze the active compounds of HZ and predict their targets and signaling pathways in UC. Molecular docking assessed binding affinities between key compounds and protein targets, identifying core bioactive compounds (Lin et al. 2024; Ct et al. 2024). To validate these predictions, we conducted in vivo experiments with a DSS‐induced ulcerative colitis mouse model and in vitro assays using IEC‐6 and T84 cell lines. Serum pharmacochemistry and in vivo tissue protein quantification investigated drug absorption, metabolism, and interactions relevant to bioavailability and efficacy. Immunofluorescence staining and flow cytometry provided further insight into molecular mechanisms, while integrated pathway analyses including IPA elucidated the biological pathways modulated by HZ. Together, these multidisciplinary approaches offer a comprehensive understanding of the active components and mechanisms behind HZ's therapeutic effects on ulcerative colitis, highlighting its potential in functional food applications.

## 2 Materials and Methods

### 2.1 Collection of HZ Active Compounds and Targets in UC Treatment

We conducted a comprehensive exploration and retrieval of compounds associated with HZ, extensively searching through PubMed (https://pubmed.ncbi.nlm.nih.gov/), Science Direct (https://www.sciencedirect.com/), and the Taiwan Electronic Theses and Dissertations System (https://ndltd.ncl.edu.tw/). We recorded the extraction methods and measurement tools relevant to these compounds during this process. Simultaneously, we compiled the identified compounds from various journals into the Traditional Chinese Medicine System Pharmacology Database 2.3 (https://tcmsp‐e.com/tcmsp.php) for screening based on conditions including an oral bioavailability value of ≥ 30%, drug‐likeness value of ≥ 0.18, and drug half‐life value of ≥ 4 h (Lin et al. 2024; Ct et al. 2024). Further predictions of compound‐related target proteins were made using the ChEMBL database. Selection criteria included confidence levels greater than 80%, activity thresholds exceeding 6, and species specificity set to *Homo sapiens* . The UniProt database (http://www.uniprot.org) was utilized for translating these proteins into gene symbols, and the GeneCards database (https://www.genecards.org/) was employed to search for genes related to ulcerative colitis (UC). Additionally, we consulted the Therapeutic Target Database (TTD, http://db.idrblab.net/ttd/) and the Comparative Toxicogenomic Database (CTD, http://ctdbase.org/) to supplement UC‐related targets. We cross‐aligned target genes induced by active compounds in HZ with UC‐related genes to identify the core target genes regulated by HZ in treating UC. Finally, a Venn diagram was generated to illustrate the distribution of target genes, providing insights into the specific activities of HZ.

### 2.2 Construction and Topological Analysis of Components‐Compounds‐Targets (CCT) Network and Protein–Protein Interaction (PPI) Network

To comprehensively understand the molecular mechanisms, we constructed the CCT and PPI network graphs using Cytoscape 3.9.1 software (Cytoscape Team, USA), setting the topological parameters of nodes, including betweenness centrality (BC), closeness centrality (CC), and degree centrality. We used the highest topological parameter levels as the primary criteria for identifying the critical compounds in HZ extracts. The 99 core target genes of HZ for UC were input into the STRING database 11.5 (https://string‐db.org/) to conduct PPI analysis. We selected PPI data with a confidence score higher than 0.7 to construct the protein interaction network, ensuring both accuracy and quantity of PPI. Based on this criterion, seven out of the 99 target genes were excluded from the PPI network due to a lack of connectivity. Finally, we used degree ≥ 28, BC ≥ 0.02, and CC ≥ 0.5 as screening criteria to identify the “key protein targets.”

### 2.3 Function Enrichment Analysis of Gene Ontology (GO) and Kyoto Encyclopedia of Genes and Genomes (KEGG) Pathway

The 99 key protein targets were submitted to the Database for Annotation, Visualization, and Integrated Discovery (DAVID) bioinformatics resources (https://david.ncifcrf.gov/) for analysis of Gene Ontology (GO) function enrichment and KEGG pathway enrichment. GO terms and KEGG pathways with a *p*‐value of less than 0.05 were considered statistically significant and valid.

### 2.4 Drugs and Reagents

Monoclonal antibodies to CD3 were purchased from Santa Cruz Biotechnology (Santa Cruz, CA; Cat# sc‐20,047). Polyclonal antibodies to TLR4 (Cell Signaling Technology; Cat# 12258) and NF‐κB (Cell Signaling Technology; Cat# 8242) were also obtained from Cell Signaling Technology. Unless otherwise specified, all other reagents were sourced from Sigma‐Aldrich (MO, USA) or Fluka (Buchs, Switzerland). Dextran sulfate sodium (DSS, CAS: 9011‐18‐1), with a molecular weight of 40,000, was purchased from MP Biomedical (Alfa Aesar, Ward Hill, MA; Cat# 311291).

### 2.5 HZ Extracts Preparation

Dried specimens of HZ were purchased from a reputable local herbal material vendor in Kaohsiung, Taiwan. Upon acquisition, the materials were sent to Chuang Song Zong Pharmaceutical Co. Ltd.—a GMP‐certified pharmaceutical facility in Kaohsiung officially recognized by the Taiwan Ministry of Health and Welfare—for authentication and safety evaluation. Botanical identification was conducted by a medicinal plant taxonomy expert from the School of Pharmacy, Kaohsiung Medical University, based on the original morphological description by Hooker (Genera Filicum, 1842, pl. 47) and the first record for Taiwan documented in Flora of Taiwan (DeVol, 1975, 1:67, pl. 17). According to the Taiwan Plant Information Integrated Query System (TAI2), HZ has no recorded synonyms or vernacular names. For chemical authentication, both water and ethanol extracts were analyzed using thin‐layer chromatography (TLC), following pharmacopeial protocols. The observed Rf values and spot colors matched those of authenticated reference standards, confirming the quality and authenticity of the material. The extraction process used distilled water as the solvent. The herbal material was boiled and extracted three times to maximize yield. The combined extract was filtered, concentrated under reduced pressure to obtain a concentrated plaster, and subsequently freeze‐dried to yield a fine powder. This powder was diluted with distilled water to the required experimental concentrations and stored at 4°C until use. All plant experiments and protocols adhered to institutional, national, and international ethical and legal guidelines.

### 2.6 Animals and Cell Lines

This study utilized 25 female 8‐week‐old C57BL/6 mice, which are considered suitable experimental subjects due to their gentler nature, relatively lower physiological needs, and easier breeding management. The mice underwent a one‐week acclimation period before the commencement of experiments. They were housed under standard conditions, including a 12‐h dark/light cycle, with unrestricted access to food and water. The mice weighed between 20 and 22 g and were initially divided into five groups (*n* = 5 mice per group). All in vivo experiments adhered to conditions approved by the Institutional Animal Care and Use Committee (IACUC) (Approval number: IACUC‐ISU‐102025) and complied with animal research assessment and accreditation standards, including the principles of replacement, reduction, and refinement. Blood collection was performed by trained personnel using aseptic techniques and methods designed to ensure safety, accuracy, and minimal distress to the animals. Approximately 100–200 μL of blood was drawn per sampling session, primarily via the submandibular vein. Sampling volumes were carefully controlled to maintain animal welfare. Collected blood samples were immediately stored on ice and subsequently processed; serum or plasma samples were aliquoted and stored at −80°C until further analysis. Furthermore, we confirm that all experiments involving our experimental animals have been conducted and reported according to the ARRIVE guidelines (https://arriveguidelines.org). Mice euthanasia was carried out through carbon dioxide asphyxiation. Intestinal epithelial cells (IEC‐6) were cultivated in Dulbecco's Modified Eagle Medium (DMEM, Cellgro, Herndon, VA, USA, catalog number: 10‐013‐CV) containing 4.5 g/L glucose and 10% fetal bovine serum (HyClone, Logan, UT, USA, catalog number: SH30070.03). T84 cells were grown in a 1:1 mixture of DMEM and Ham's F‐12 medium (Cellgro, catalog number: 10‐092‐CV) containing 10% fetal bovine serum. We hereby disclose that the IEC and T84 cell lines used in our research investigations were procured from AddexBio (catalog numbers: P0021001 and C0009008).

### 2.7 Cell Viability Assay

T84 and IEC‐6 cells were seeded in 96‐well plates (Corning Glass Works, Corning, NY, USA; Cat# 3596) at a density of 1 × 10^5^ cells/well and cultured overnight at 37°C in an atmosphere of 5% CO_2_ and 95% O_2_. The cells were then treated for 24 h with a serum‐free medium containing different concentrations of TC (10%, 20%, 30%, 40%, 50%). Cell viability was assessed using MTS (Promega, Madison, WI, USA; Cat# G3581).

### 2.8 Experimental Design

We used 3% (*w*/*v*) DSS for five consecutive days (Day 1 to Day 5) to induce UC in mice (Wu et al. 2014). The animals were randomly assigned to five groups: a standard control group, a DSS‐induced colitis model group, and three DSS + HZ water extract treatment groups receiving 0.1 g/kg, 1 g/kg, or 2 g/kg doses of HZ. In our pilot dose‐finding study using the DSS‐induced UC model, the IC_50_ of the HZ extract was estimated at approximately 0.85 g/kg, which is substantially higher than the effective doses reported in previous asthma model studies (Wu, Huang, et al. 2017). This discrepancy likely reflects the pathophysiological differences between respiratory and gastrointestinal disease models, particularly the need for higher local concentrations in the colon to achieve therapeutic efficacy. To capture a dose–response relationship while remaining within a safe range, we selected a graded dosing scheme: 0.1 g/kg (low dose, below IC_50_ to assess minimal response), 1 g/kg (medium dose, slightly above IC_50_ to evaluate therapeutic efficacy), and 2 g/kg (high dose, near the safety threshold to explore maximal effects). This strategy aligns with OECD acute oral toxicity guideline TG 423, which recommends limit tests up to 2000 mg/kg (~2 g/kg) when prior data indicate low toxicity, and is further supported by botanical extract studies demonstrating safe repeated dosing at 1–2 g/kg in rodent models. The HZ powder was dissolved in distilled water to prepare the respective concentrations, and test samples were administered orally once daily by gavage from Day 6 to Day 13 (a seven‐day treatment course), as illustrated in Figure S1. The one‐week treatment period was selected based on established DSS‐induced UC protocols, which typically apply a 7‐day intervention to assess therapeutic efficacy while minimizing prolonged exposure that may lead to toxicity (Ren et al. 2023). After euthanasia, the colon was extracted and thoroughly cleaned of mesentery tissue, vessels, and fat, and its length was measured. The colon and small intestine were removed and rinsed with 1X PBS (Gibco, Catalog no. 10010049). For histological examination, a 1 cm segment from the distal colon was fixed in 10% formalin (Sigma‐Aldrich, Catalog no. HT501128) for 24 h at room temperature. The fixed tissue was then embedded in paraffin and sectioned into 4‐μm thick slices using a microtome. Hematoxylin and eosin staining (H&E, Sigma‐Aldrich, Catalog no. HT110216) was performed by first deparaffinizing the tissue sections in xylene, followed by rehydration through a graded ethanol series (100%, 95%, 70%, and distilled water). The sections were stained with hematoxylin for 5–10 min, rinsed with tap water, counterstained with eosin for 1–2 min, then dehydrated, cleared in xylene, and mounted with a coverslip. Periodic acid‐Schiff staining (PAS, Sigma‐Aldrich, Catalog no. 395B‐1KT) was performed similarly. After deparaffinizing and rehydrating, the sections were treated with 1% periodic acid for 10 min, rinsed in distilled water, stained with Schiff reagent for 15 min, then rinsed, dehydrated, cleared in xylene, and mounted with a coverslip. These procedures allowed for the evaluation of tissue morphology and histopathological changes. For blood collection, we employed cardiac punctures. Specifically, after anesthetizing the mice, a sterile needle was used to puncture the left ventricle of the heart to collect 1–2 mL of blood. The blood was immediately placed into EDTA‐coated tubes to prevent coagulation. Subsequently, the blood was centrifuged using an Eppendorf 5702 centrifuge (Eppendorf, Catalog no. 5702) at 3000 rpm for 10 min to separate the plasma. The plasma layer was carefully transferred into new sterile tubes and stored at −80°C until further analysis. We utilized the histologic inflammatory activity index to assess the improvement of inflammation induced by DSS and evaluated the status of inflammatory cell infiltration through histological analysis. According to the Gupta histologic index, inactive or quiescent status showed no neutrophil infiltration in the epithelium; mild activity indicated neutrophil infiltration in less than 50% of crypts or cross‐sections without ulcers or erosions; moderate activity showed neutrophil infiltration in 50% or more of crypts or cross‐sections without ulcers or erosions; severe activity showed erosion or ulceration regardless of other features (Gupta et al. 2007; Marchal Bressenot et al. 2015; Bressenot et al. 2015). In the in vitro arm, IEC‐6 and T84 monolayers were exposed to DSS at 2% (*w*/*v*) for 12 h to induce an epithelial inflammatory/injury phenotype. The 2% range is widely used to model UC‐like epithelial disruption in human intestinal lines (e.g., Caco‐2/HT‐29), and permeability changes typically peak within a 6–12 h window after DSS exposure; we therefore selected 12 h as a robust yet non‐lethal induction period for both IEC‐6 and T84, consistent with prior reports of effective barrier disruption at ~2% and within this timeframe (Wang et al. 2021). Model establishment was pre‐confirmed by ELISA showing a significant rise of TNF‐α and IL‐6 in culture supernatants versus untreated controls, after which HZ interventions were initiated.

### 2.9 Assessment of Colon and Intestinal Damage

Daily monitoring and recording included observations on body weight, stool consistency, and the presence of gross bleeding. The Disease Activity Index (DAI) was calculated based on score changes, considering parameters such as body weight loss index (0 = none; 1 = 1%–3%; 2 = 3%–6%; 3 = 6%–9%; 4 = over 9%), stool consistency index (0 = normal; 2 = loose stools; 3 = diarrhea), and gross bleeding index (0 = average; 2 = hemoccult; 3 = gross bleeding), following previously established criteria. On day 13, animals were euthanized, and the colons were harvested (Smith et al. 2007). The length of the colon was measured from the cecum to the anal verge. The sections were stained with H&E to assess general histological changes, such as tissue structure, inflammation, and cell damage. PAS staining was used to evaluate mucosal ulceration and mixed inflammatory cell infiltrates, highlighting mucins and glycogen to identify mucosal damage and inflammation. This combined approach allows for a comprehensive analysis of tissue morphology and inflammatory responses.

### 2.10 Measurement of Systemic Cytokine Levels

We measured the concentrations of TNF‐α and IL‐6 in mice's plasma and colon and small intestine tissues. The concentrations of TNF‐α and IL‐6 in the plasma were determined using commercial ELISA kits (BioLegend, San Diego, CA, USA, TNF‐α kit catalog number: 430907; IL‐6 kit catalog number: 431307), following the manufacturer's instructions. The colon and small intestine tissues were rinsed with 1X phosphate‐buffered saline (PBS, pH 7.4), quickly immersed in liquid nitrogen (−196°C) for freezing, and stored at −80°C for further analysis. Subsequently, these tissues were homogenized in PBS containing 1 mM phenylmethylsulfonyl fluoride (PMSF, Sigma‐Aldrich, catalog number: 93482) and 10 μg/mL of aprotinin (Sigma‐Aldrich, catalog number: A6279), leupeptin (Sigma‐Aldrich, catalog number: L2884), and pepstatin A (Sigma‐Aldrich, catalog number: P5318) were added per milliliter. The homogenates were centrifuged at 12,000 rpm for 15 min at 4°C. The concentrations of IL‐6 and TNF‐α in the tissues were also measured using commercial ELISA kits, and the data were expressed as pg/mL of cytokine per milligram of tissue protein.

### 2.11 Investigation of CD3 and TLR4 Expression by Immunofluorescence

We conducted an immunofluorescence analysis to evaluate CD3 and TLR4 expressions in formalin‐fixed, paraffin‐embedded tissue sections. Sections (3‐μm thick) were deparaffinized in xylene (Sigma‐Aldrich, Cat# 534056) and rehydrated through graded ethanol (70%, 80%, 95%, 100%; Sigma‐Aldrich) for 5 min per step. Antigen retrieval was performed in 10 mM citrate buffer (pH 6.0; Sigma‐Aldrich, Cat# C9999) using a microwave at medium power (600 W) for 5 min, then cooling. Sections were blocked with 1% BSA in PBS (Sigma‐Aldrich, Cat# A9647; Gibco, Cat# 10010023) for 1 h at room temperature. Primary antibodies, monoclonal anti‐CD3 (Santa Cruz, Cat# sc‐20047) and polyclonal anti‐TLR4 (Santa Cruz, Cat# sc‐293072), both diluted 1:100 in 1% BSA‐PBS, were applied for 1 h at room temperature. After three PBS washes, sections were incubated with Rhodamine‐conjugated secondary antibody (IMGENEX, Cat# IMG‐579) diluted 1:300 in 1% BSA‐PBS for 1 h in the dark, followed by further PBS washes. Negative controls (without primary antibodies) and positive controls (tissues known to express CD3 and TLR4) were included to confirm specificity. Antibody concentrations and conditions were optimized to ensure a high signal‐to‐noise ratio. Finally, sections were mounted with Fluoromount‐G (Thermo Fisher, Cat# 00‐4958‐02) and imaged using a Nikon Eclipse 4000 fluorescence microscope. Fluorescence intensity was analyzed, and experiments were repeated to ensure reproducibility.

### 2.12 Western Blot Analysis

Protein samples were subjected to 8% to 10% polyacrylamide gel electrophoresis (SDS‐PAGE) and then transferred to nitrocellulose membranes (Whatman, Buckinghamshire, England; Cat# 10401396). The membranes were blocked for 1 h in Tris‐buffered saline (TBS) containing 0.1% Tween and 5% nonfat skim milk. Subsequently, the membranes were incubated overnight at 4°C with primary antibodies, including polyclonal anti‐TLR4 antibody (Santa Cruz Biotechnology, Santa Cruz, CA, USA; Cat# sc‐293072) and polyclonal anti‐NF‐κB antibody (Santa Cruz Biotechnology, Santa Cruz, CA, USA; Cat# sc‐8008). Afterward, the membranes were washed with TBS‐T and incubated with HRP‐conjugated secondary antibodies (diluted 1:1000) for 1 h at room temperature. Visualization of bands was achieved using the ECL Plus chemiluminescence detection system (GE Healthcare, Pittsburgh, PA; Cat# RPN2132), and band densities were determined with a Canon CanoScan 4400F scanner and ImageJ densitometry software.

### 2.13 Statistical Analysis

The data are presented as means ± standard error of the mean (SEM) and were analyzed using SPSS 13.0 software (SPSS Inc., Chicago, IL, USA) and graphically visualized with GraphPad Prism 9 software (LLC, California, USA). Statistical analysis was performed using the student's t‐test for comparisons between two groups and one‐way analysis of variance (ANOVA) for comparisons among multiple groups. Prior to ANOVA, Levene's Test was conducted to verify the homogeneity of variances across groups. When ANOVA revealed significant differences (*p* < 0.05), post hoc multiple comparisons were performed using the Least Significant Difference (LSD) test to identify which specific groups differed significantly. Statistical significance was set at *p* < 0.05. Significant differences are clearly indicated in figures and tables using appropriate symbols (e.g., *, **).

### 2.14 Molecular Docking Analysis

Utilizing the QIAGEN Ingenuity Pathway Analysis (IPA) software, we simulated to elucidate potential pathways involving TLR4 and NF‐κB proteins in the pathophysiological mechanisms of active UC (Krämer et al. 2014). Additionally, we investigated the associated upstream and downstream proteins within these pathways. Subsequently, the identified proteins, assumed to act as receptors, were integrated into a molecular docking analysis to assess their molecular affinity with the ligand HZ compounds quantitatively. Molecular docking was analyzed using AutoDock 4.2 software, which employs the Lamarckian genetic algorithm. All the three‐dimensional structures of target proteins in the TLR4/NF‐κB pathway were downloaded from the RCSB PDB database (https://www.Rcsb.org/), including TLR4 (pdb id: 4G8A) (Ohto et al. 2012), RELA (pdb id: 1IKN) (Huxford et al. 1998), NFKBIA (pdb id: 1NFI) (Jacobs and Harrison 1998), MyD88 (pdb id: 4DOM) (Snyder et al. 2013), IRAK4 (pdb id: 6O8U) (Bryan et al. 2019), TRAF4 (pdb id: 3ZJB) (Rousseau et al. 2013), MAP3K7 (pdb id: 5JGA) (Muraoka et al. 2016) and IKBKB (pdb id: 4KIK) (Liu et al. 2013). We meticulously chose target protein models sourced from the RCSB PDB database, acquired through X‐ray crystallography techniques to guarantee the active conformation. Furthermore, we exclusively assessed protein models with a resolution of less than 3 Å to ensure the requisite crystal quality (Read et al. 2011). An additional screening criterion was applied for X‐ray crystallography models, stipulating that the *R*‐value free should be less than 0.3. This additional criterion enhanced the validation of the selected models' accuracy (Kleywegt and Alwyn Jones 1997). The parameters for pre‐docking preparation will be detailed in Table S1. Prior to conducting the molecular docking experiments, extraneous molecules such as antibody fragments, glycerol, H2O, free ions, inositol‐1,3,4,5‐tetrakisphosphate, and S‐sulfinylation, and so forth, were removed from the selected target protein models. Meanwhile, the region corresponding to the orthostatic site of the original model was retained and substituted with our active compounds for the subsequent docking procedure. (Ct et al. 2024) All docking simulations adhered to default settings, producing a maximum of nine binding modes. The docking result with the highest absolute value of binding energy was selected as the most favorable representation of the receptor‐ligand interaction simulation.

## 3 Results

### 3.1 Potential Target Genes and Compounds Among HZ and UC

We systematically compiled and analyzed 18 distinct compounds derived from HZ identified in 11 relevant studies (18–25), detailing the corresponding extraction methods and mass spectrometry detection techniques, as summarized in Table S2 (Wu, Kao, et al. 2017; Liou et al. 2017; Huang et al. 2017; Lin et al. 2023; Wu et al. 2014; Liu, Ho, et al. 2023; Chan et al. 2013; Wu, Huang, et al. 2017). These compounds include various plant sterols (e.g., galactitol, stigmasterol, β‐sitosterol), fatty acids (palmitic acid, stearic acid), flavonoids such as quercetin, and several Ugonin derivatives (Ugonin J, Ugonin K, Ugonin L, Ugonin M, Ugonin N, Ugonin O, Ugonin P, Ugonin Q, Ugonin R, Ugonin S, Ugonin T, and Ugonstilbene A). Various extraction and isolation methods were also documented. To analyze potential target genes, we identified 917 genes associated with UC from the GeneCards, TTD, and CTD databases. Additionally, 257 genes related to the 18 HZ compounds were identified using the TCMSP and ChEMBL databases. A Venn diagram highlighted 99 potential target genes between HZ and UC (see Figure S2A). A summary of the active compounds and their corresponding potential target genes is shown in Table S3, while the structural formulas of each active compound are depicted in Figure S2B.

### 3.2 Topological Analysis of CCT and PPI Network

**FIGURE 1:**
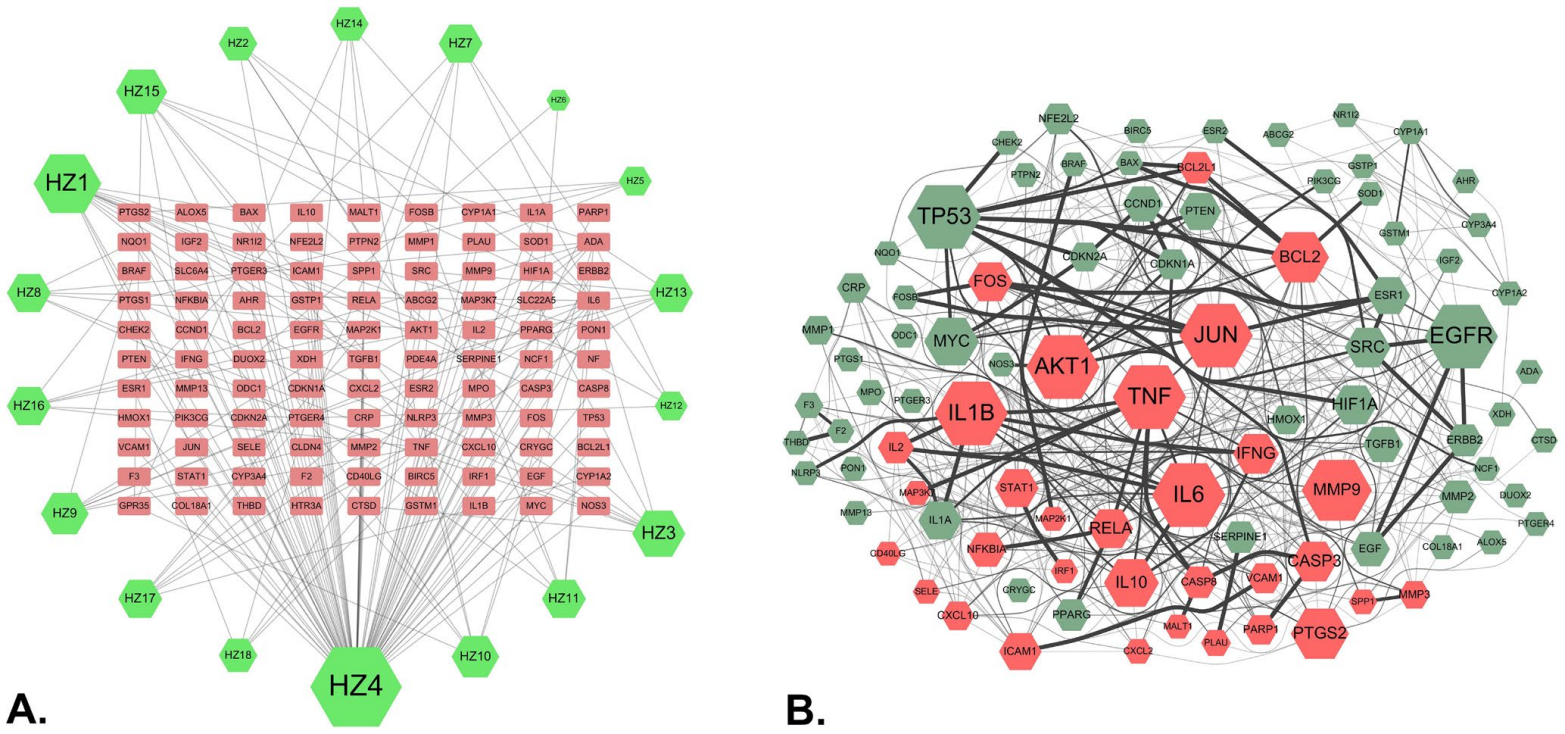
(A) Components‐compounds‐targets network of HZ. The green hexagons represent the 18 active compounds derived from HZ, while the pink circles denote the 99 potential target genes. The nodes' sizes indicate the active compounds' DC, with other target gene nodes displayed in a standardized size. (B) The interactive PPI network of HZ target proteins associated with UC. The size of the nodes is positively correlated with the node degree criterion. The varying width and transparency of the edges, determined by the combined score of two target proteins, illustrate the extent of their interactions. Additionally, proteins with similar biological functionalities are grouped into distinct clusters.

In Figure 1A, the CCT network shows that the size of the green hexagons, which is positively correlated with the Degree, represents the association patterns between different compounds and target proteins. Table S4 details the arrangement of active compounds based on topological parameters. Following this, a PPI network analysis incorporated 99 target protein molecules. Notably, seven target proteins (NF, SLC6A4, SLC22A5, CLDN4, GPR35, HTR3A, and PDE4A) did not establish interactions with the remaining 92 target proteins, as their combined scores fell below the selection threshold of 0.7. As illustrated in Figure 1B, the resulting network consists of 92 nodes (representing target proteins) and 769 edges (representing interactions). Two distinct clusters, marked in pink and green, show a significant functional association with specific inflammatory pathways closely related to UC mechanisms, while others correspond to non‐specific pathways. These specific inflammatory pathways, such as the NF‐κB pathway, Toll‐like receptor pathway, and TNFα signaling pathway, closely align with the results from the subsequent KEGG analysis. Based on network property criteria (Degree ≥ 28, BC ≥ 0.02, CC ≥ 0.5), 15 core target proteins were identified in the PPI network analysis. (Table S5) Subsequent MCC analysis further validated and confirmed the accuracy of these 15 core target proteins. (Figure S6) Furthermore, the width and transparency of the edges, determined by the combined score, indicate that target proteins within the same protein cluster interact not only, but also there are significant interaction patterns between different protein clusters (Table S6). These findings suggest that in the therapeutic mechanism of HZ, shared regulatory pathways may extend beyond a single major pathway, highlighting the multi‐compound nature of TCM.

### 3.3 Functional and Pathway Enrichment Analysis

**FIGURE 2:**
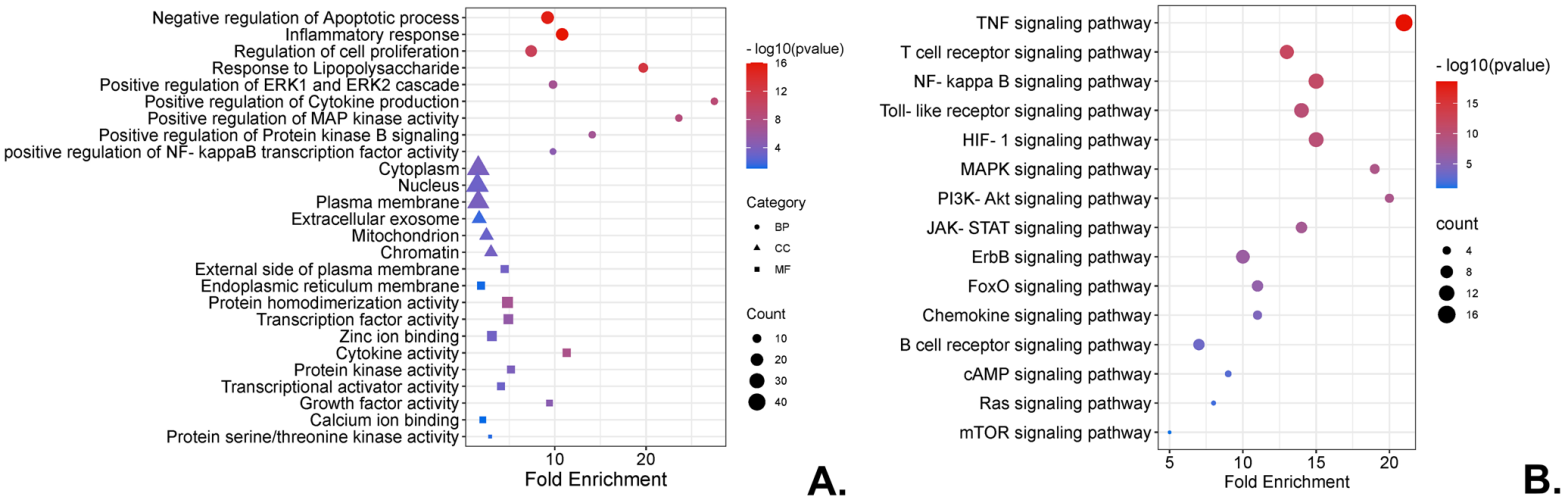
(A) The GO enrichment annotation of target genes shows the functional distribution across BP, CC, and MF terms. These terms were ranked based on gene counts, *p*‐values, and fold enrichment. Different shapes indicate distinct GO terms. The *y*‐axis represents gene functional classifications, while the *x*‐axis shows the corresponding fold enrichment values. There is a positive correlation between node size and the number of target genes, and the color gradient corresponds to the negative logarithm of the *p*‐value, with brighter red shades indicating higher values. (B) KEGG enrichment analysis of target genes. The color scale reflects varying thresholds of adjusted *p*‐values, while the dot sizes represent the gene count for each pathway. The *y*‐axis categorizes the KEGG pathways, and the *x*‐axis displays the level of fold enrichment.

In Figure 2A and Table S5, we conducted GO functional enrichment analysis, which identified several significantly enriched BP, including the inflammatory response, lipopolysaccharide response, regulation of cell proliferation, apoptotic processes, cytokine production, and MAP kinase activity. Among the key pro‐inflammatory mediators, interleukins such as IL6, IL1β, and IL10, along with TNFα, play pivotal roles in driving the inflammatory response. In addition, regulatory proteins like JUN, EGFR, RELA, ESR1, PTGS2 (COX‐2), MMP9, TP53, and NFKBIA play integral roles in modulating inflammation by regulating processes such as cell proliferation, apoptosis, and cytokine production. The CC annotations indicate enrichment in the cytoplasm, nucleus, mitochondrion, and chromatin. Proteins located in the cytoplasm, including IL6, TNFα, IL1β, JUN, EGFR, RELA, PTGS2, NFKBIA, MYC, AKT1, and IL10, primarily participate in intracellular processes such as signal transduction, regulation of cell proliferation, and inflammatory responses. Under specific conditions, EGFR and RELA translocate to the nucleus to perform corresponding regulatory functions. Proteins in the nucleus, such as ESR1, TP53, MYC, and IL10, are mainly involved in transcriptional processes. Additionally, proteins on the cell membrane, including EGFR and AKT1, participate in membrane‐related signal transduction, while PTGS2 functions within the endoplasmic reticulum. MMP9 acts on the extracellular matrix. MF annotations reveal activities related to cytokine activity, protein kinase activity, transcriptional activator activity, and protein homodimerization, among others. JUN is a crucial transcriptional activator that influences gene expression. EGFR and AKT1 contribute to complex cellular signal transduction through their protein kinase activity, while ESR1 and TP53, as transcriptional activators, regulate estrogen‐responsive genes, impacting the cell cycle and immune response. PTGS2 participates in specific cell signal transduction and inflammation through its kinase activity. MMP9 is essential for substrate degradation via its proteinase activity. RELA and NFKBIA, which are involved in NF‐κB signaling, modulate immune responses and inflammation. Finally, BCL2, JUN, and MYC use protein homodimerization, influencing cell survival and apoptosis.

The KEGG signaling pathway enrichment analysis identified 56 pathways, with the top 15 displayed in Figure 2B and Table S7. The pathway with the lowest *p*‐value is the most relevant to the hypothesized mechanism of HZ treatment. Notable pathways include the Toll‐like receptor signaling pathway, NF‐κB signaling pathway, T cell receptor signaling, TNFα signaling, and others. These findings informed the selection of protein markers—such as TNFα, IL6, CD3, TLR4, and the NF‐κB complex—for our animal experiments, enabling a more detailed validation based on these results.

### 3.4 Mucosal Protective Effect of HZ Water Extract in DSS‐Induced Colitis Mice

**FIGURE 3:**
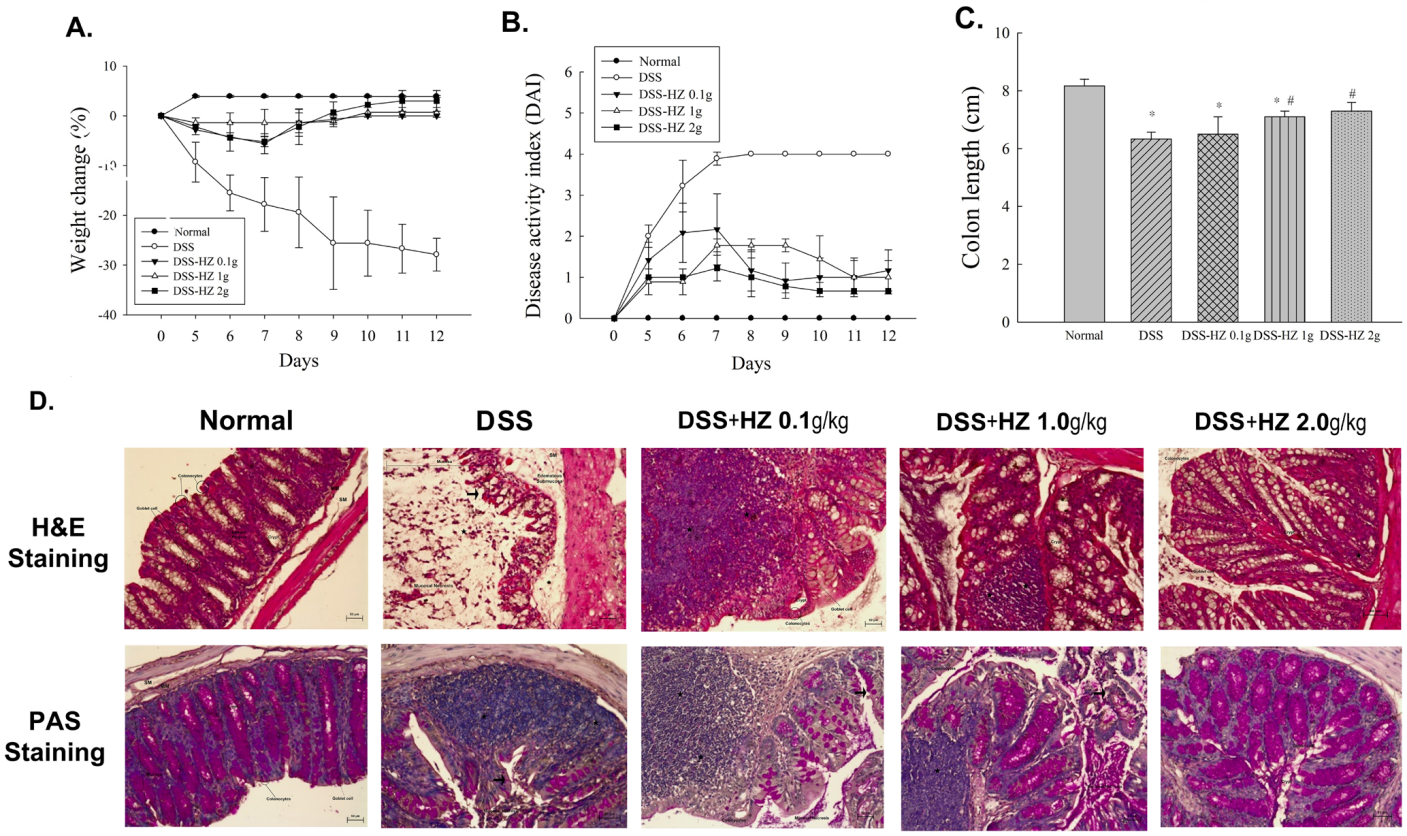
(A) Weight loss monitoring of mice. (B) Average DAI score of mice. (C) The colon length of each group on day 12 is presented. The data represent four experiments, with scale bars indicating 1 cm. Results are expressed as means ± standard error (*n* = 5). *p < 0.01 versus control mice; #*p* < 0.05 versus DSS‐treated mice. (D) Representative H&E and PAS staining of colon tissue (magnification, ×200) is shown. Abbreviations used in microscopic images include colonocytes, goblet cells, crypts, lamina propria, mucosa, submucosa (SM), and muscularis mucosa (MM). Key highlights include the disappearance of colonocytes (bold arrows) and the infiltration of inflammatory substances and neutrophils (stars).

DSS ingestion induced severe colitis in mice, evidenced by significant weight loss, diarrhea or loose stools, and visible blood in the feces, leading to a marked increase in DAI scores. DAI scores in the DSS group steadily rose until day 5. From days 6 to 12, we administered water extracts of HZ at doses of 0.1, 1, and 2 g/kg to the mice. As shown in Figure 3A, the water extracts of HZ (0.1, 1, and 2 g/kg) effectively mitigated weight loss during DSS‐induced colitis. Moreover, the HZ water extracts produced a dose‐dependent reduction in DAI scores compared to the DSS group, with a significant protective effect observed on day 12 (Figure 3B). We also evaluated the effect of the HZ water extract on colon length, a parameter inversely correlated with colitis severity. Colon shortening is a common consequence of inflammation and tissue damage in colitis, reflecting the degree of mucosal injury and repair. As shown in Figure 3C, the HZ water extract groups (1 and 2 g/kg) significantly prevented colon shortening compared to the DSS group (*p* < 0.05).

Histological analysis using H&E staining revealed various characteristics of mucosal damage in the colons of mice subjected to DSS treatment, including inflammatory cell infiltration (penetrating the entire mucosal and submucosal layers), submucosal edema, loss of glandular crypts, and damage to goblet cells and colonic epithelial cells. These findings align with the typical histological features of chronic active colitis observed in untreated UC, where epithelial injury mediated by neutrophils manifests as cryptitis, accumulation of neutrophils within crypt lumens, and mucosal ulceration (DeRoche et al. 2014). Chronic changes include distorted crypt architecture, shortening or complete loss of crypts, and basal neutrophil infiltration, often accompanied by edematous submucosa (Theodossi et al. 1994). However, in the DSS‐HZ 2 g/kg group, significant reductions in tissue alterations were observed compared to the DSS group. This group exhibited regenerated glandular crypts, abundant goblet cells, a more intact epithelial structure with newly formed colonic cells, reduced neutrophil infiltration, and a structure resembling the standard control group. In the DSS‐HZ 0.1 g/kg group, while neutrophil infiltration was more extensive, the degree of mucosal necrosis was milder compared to the DSS group, and the mucosal structure remained visible. The DSS‐HZ 1 g/kg group showed extremely mild mucosal damage but still exhibited neutrophil infiltration (Xu et al. 2020).

Additionally, PAS staining provided a more precise visualization of goblet cells in the colonic mucosa of each group. The extent of neutrophil infiltration was more pronounced, with consistent results observed across both staining modalities. The protective effects of HZ on DSS‐induced colonic mucosal damage appeared to be dose‐dependent, with particularly favorable therapeutic outcomes observed at the 2 g/kg dose (Figure 3D).

### 3.5 HZ Modulates Colonic and Serum Proinflammatory Cytokine Production

**FIGURE 4:**
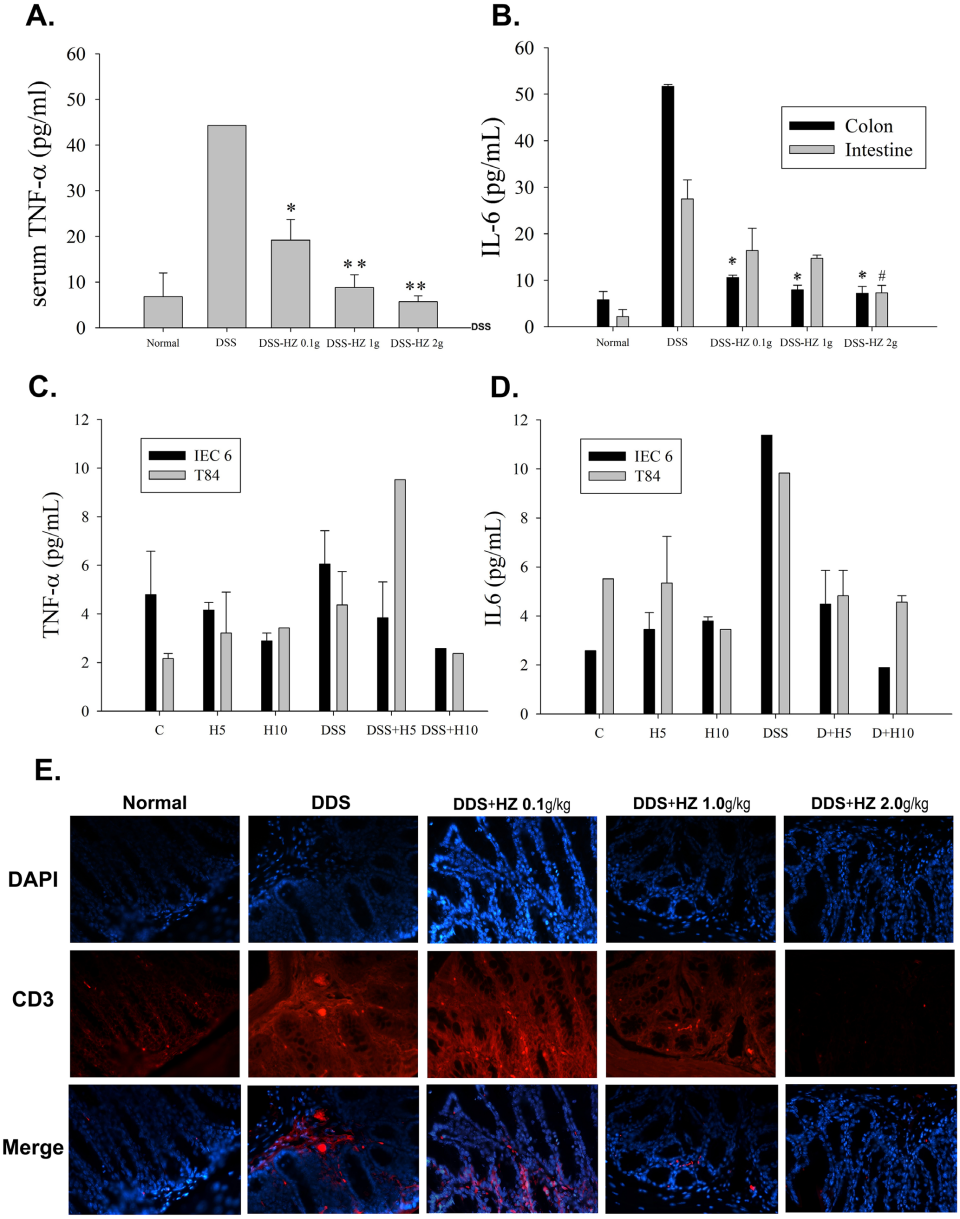
(A) TNF‐α concentration in the plasma of mice from all groups. *p < 0.05 versus DSS mice; **p < 0.01 versus DSS mice. (B) IL‐6 concentration in the colon and intestinal tissues of mice from all groups. *p < 0.01 (colon tissue); #*p* < 0.05 (intestinal tissue). (C) TNF‐α concentration in the medium of IEC‐6 and T84 cells from all groups. (D) IL‐6 concentration in the medium of IEC‐6 and T84 cells from all groups. (E) Immunofluorescent staining with CD3 in colon tissues from mice. Red staining indicates CD3, while DAPI (blue) is a nuclear stain. All images are magnified ×400.

To confirm the inhibitory effects of HZ on systemic cytokine levels following DSS induction and validate the results obtained from the PPI network, we measured the levels of the inflammatory cytokines TNF‐α and IL‐6 in mice plasma, colon, and small intestine tissues through ELISA analyses. Compared to the control group, the systemic levels of TNF‐α significantly increased in the DSS group. Treatment with HZ at 2 g/kg (5.7 ± 1.3) and 1 g/kg (8.8 ± 2.8) resulted in a significant reduction in TNF‐α expression compared to mice treated with DSS alone (44.3 ± 0.0). However, treatment with HZ at 0.1 g/kg (19.2 ± 4.5) showed a slight decrease in TNF‐α expression compared to the DSS group, although it did not reach statistical significance (Figure 4A). The IL‐6 levels in the colon tissue of the DSS group were significantly higher than those in the control group. Treatment with HZ at 2 g/kg (7.2 ± 1.5), 1 g/kg (8.0 ± 1.0), and 0.1 g/kg (10.6 ± 0.5) significantly attenuated IL‐6 expression compared to the DSS‐treated mice (51.7 ± 0.4), with all P values being less than 0.01 (Figure 4B). This effect was primarily observed in the colon tissue experimental group, while significant reductions in IL‐6 levels in the small intestine were only noted with higher concentrations of HZ treatment.

Moreover, we assessed the impact of HZ on cytokine production during DSS‐induced colitis in an in vitro study. We measured the levels of the proinflammatory cytokines TNF‐α (Figure 4C) and IL‐6 (Figure 4D) using ELISA in the IEC‐6 and T84 cell culture media. Compared to the control group, the levels of TNF‐α and IL‐6 significantly increased in the DSS group, both in IEC‐6 and T84 cell culture media. For TNF‐α expression, administration of IEC‐DSS + HZ at 5 mg/kg (3.8 ± 1.5) and IEC‐DSS + HZ at 10 mg/kg (2.6 ± 0.0) resulted in significant reductions compared to cells treated with DSS alone (6.1 ± 1.4). Similarly, T84‐DSS + HZ at 10 mg/kg (2.0 ± 0.0) showed attenuation compared to cells treated with DSS alone (4.4 ± 1.4). Regarding IL‐6 expression, treatments with IEC‐DSS + HZ at 5 mg/kg (4.5 ± 1.4) and IEC‐DSS + HZ at 10 mg/kg (1.9 ± 0.0) significantly decreased compared to cells treated with DSS alone (11.4 ± 0.0). Similarly, T84‐DSS + HZ at 5 mg/kg (4.8 ± 1.0) and T84‐DSS + HZ at 10 mg/kg (4.6 ± 0.3) showed attenuation compared to cells treated with DSS alone (9.8 ± 0.0).

### 3.6 Properties of T Cells Following HZ Treatment During DSS‐Induced Colitis

Immunofluorescence analysis revealed an increased expression of IL‐6 in CD3+ T cells from the lamina propria lymphocytes during DSS‐induced acute colitis (Figure 4E). CD3+ T lymphocytes will likely release cytokines when activated, suggesting that IL‐6 results from T lymphocyte activation (Abreu‐Martin and Targan 1996; Ma et al. 2023). Treatment with HZ at a dose of 2 g/kg significantly reduced the infiltration of IL‐6‐expressing CD3+ T cells compared to the DSS group and mice treated with lower doses. These results collectively confirm that HZ treatment downregulates the activity of CD3+ T cells in the lamina propria lymphocytes of DSS‐induced mice.

### 3.7 Analysis of NF‐κB Complex and TLR4 Expressions

UC‐associated cancer tissues exhibit overexpression of TLR4. Mice deficient in TLR4 demonstrate protective effects against DSS‐induced UC‐associated neoplasia, indicating that TLR4 promotes tumorigenesis by modulating colonic epithelial cells' innate immune signaling pathways (Pastille et al. 2021; Fukata et al. 2011). TLR4 deficiency and the loss of commensal bacteria increase tolerance to DSS‐ and radiation‐induced intestinal injury. Concurrently, phytochemicals and TCMs reduce TLR4/NF‐κB activation, thereby ameliorating the pathological manifestations of DSS‐induced UC in mouse models (Xiong et al. 2022; Ke et al. 2013). These studies highlight the complex regulatory roles of the TLR4 and NF‐κB systems in UC‐associated tumor formation and intestinal damage repair. Our KEGG analysis also revealed that HZ treatment in UC involves crucial inflammatory mechanisms, particularly the NF‐κB signaling and Toll‐like receptor signaling pathways.

**FIGURE 5:**
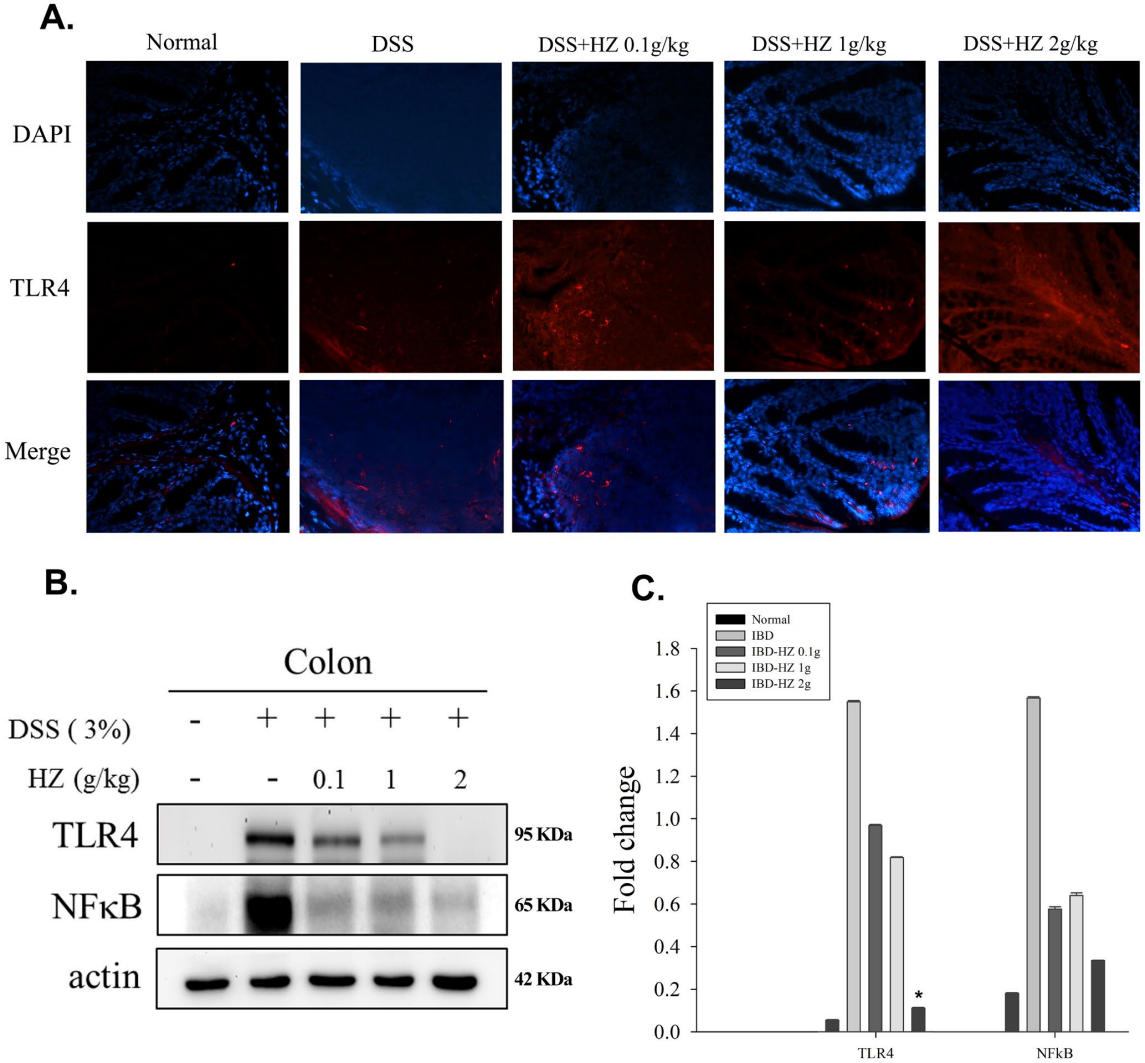
(A) Immunofluorescent staining with TLR4 in colon mucosa from mice. Immunofluorescent staining for TLR4 (red) staining was performed in the inflamed colon. Nuclei are stained with blue (DAPI); all magnifications are × 400. (B) Western blot analysis of TLR4 (95 kDa), NF‐κB p65 (RELA, 65 kDa), and Actin (42 kDa) in colon tissue. (C) The relative intensity of each band in the western blots was determined using densitometry, and the ratios were calculated. *p < 0.05 versus DSS mice in TLR4 expression.

Subsequently, we conducted immunohistochemical staining experiments on colonic tissues (Figure 5A), which demonstrated that HZ treatment reduces the expression of TLR4 protein in DSS‐induced mice, particularly in the HZ 2 g/kg group (*p < 0.05). Moreover, HZ treatment significantly inhibits TLR4 activity in a dose‐dependent manner (Figure 5B). As DSS induces TLR4 activation, we investigated the impact of HZ on subsequent NF‐κB activity, explicitly aiming to confirm its ability to reverse the elevated levels of NF‐κB p65 associated with activated TLR4. Western blot experiments revealed that all HZ treatment groups could reduce NF‐κB p65 (RELA) levels, although statistical significance was not achieved (Figure 5C). However, in vitro studies using Western blot experiments in T84 or IEC‐6 cell groups did not demonstrate significant inhibition of TLR4 and NF‐κB activity (Figure S3).

### 3.8 The Anti‐Cell Proliferative Effect of HZ Extract

**FIGURE 6:**
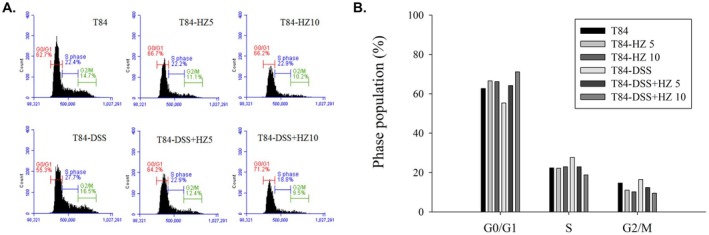
(A) Flow cytometry analysis on cell cycle of T84 treated with HZ of different concentrations for 16 h. (B) Cell cycle distribution of T84 cells.

In the pathological process of UC, excessive activation of immune cells releases inflammatory mediators such as cytokines and chemokines, which cause damage to mucosal tissues. One of the most notable changes is the alteration in the renewal rate of the epithelial layer, typically manifested by increased epithelial proliferation and an augmented thickness of the colonic mucosa (Sipos et al. 2005). When tissues are damaged, the body initiates a repair process involving cell proliferation and regeneration to restore tissue integrity. These characteristics can be observed in T84 cells. Abnormal hyperproliferation induced by DSS in T84 cells has also been documented in vitro studies. Our flow cytometry analysis in Figure 6 revealed a dose‐dependent increase in the G0/G1 cell cycle phase in DSS‐treated T84 cells after HZ treatment, indicating G0/G1 cell cycle arrest. This phenomenon helps explain HZ's anti‐inflammatory response and corroborates our GO analysis results, suggesting that HZ treatment primarily involves the biological “regulation of cell proliferation.” However, our results did not reveal a SubG1 peak, indicating that it may not be associated with the apoptotic pathway.

### 3.9 Molecular Docking Analysis of Compounds‐Targets in TLR4/NF‐κB Pathway

**FIGURE 7:**
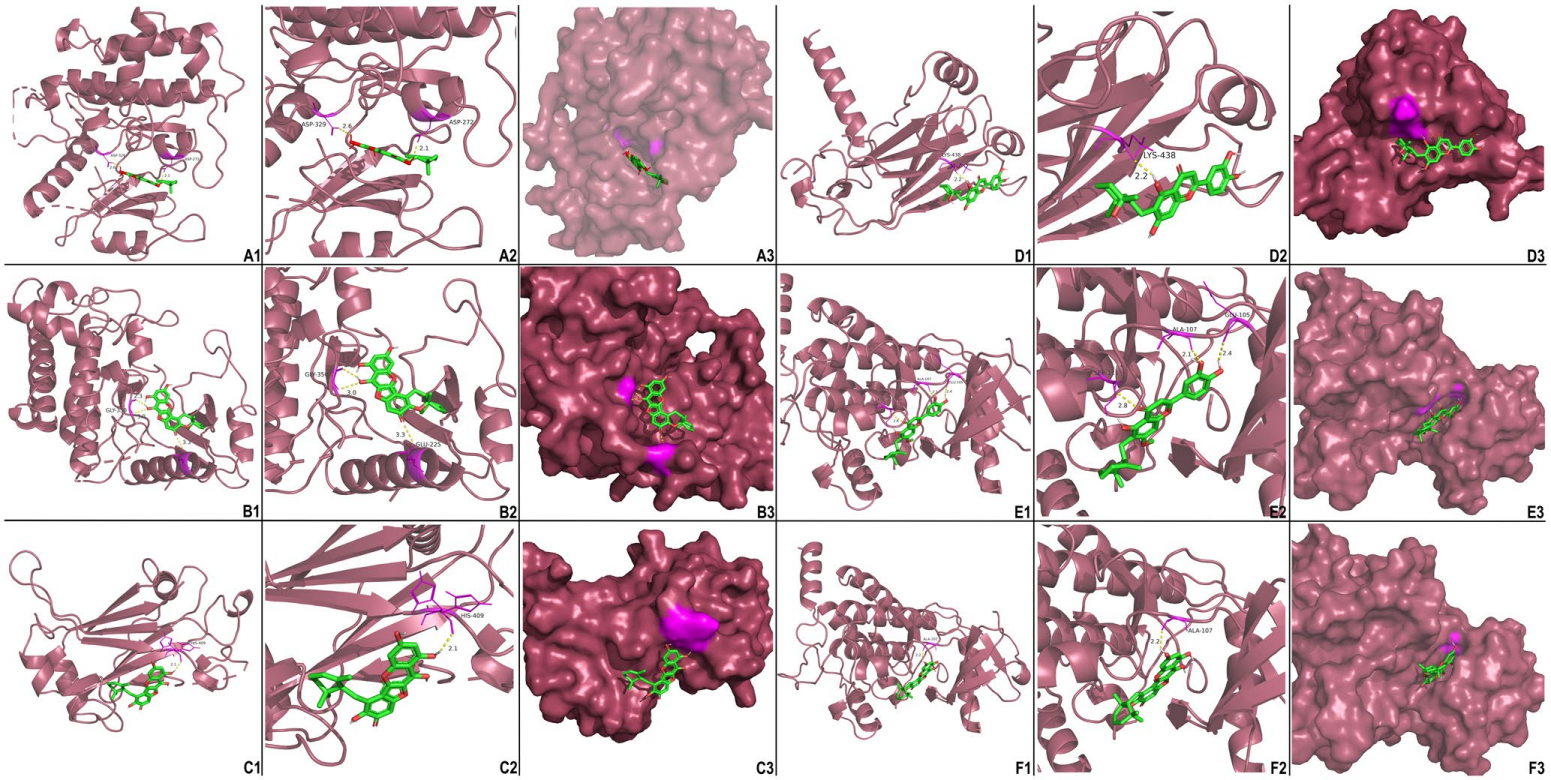
3D molecular docking visualization results of target proteins with active compounds of HZ (A) Ugonin M with MAP3K7 (5JGA). (B) Ugonin O with MAP3K7 (5JGA). (C) Ugonin M with IKBKB (4KIK). (D) Ugonin R with IKBKB (4KIK). (E) Ugonin K with RELA (1IKN). (F) Ugonin O with RELA (1IKN). Green‐red stick models represent active compounds, while the secondary structure of the proteins is depicted using dark burgundy ribbon representations. The yellow lines between active compounds and target proteins indicate the polar hydrogen bonding interactions. Additionally, surface conservation analysis highlights how receptor compounds bind to ligand proteins.

**FIGURE 8:**
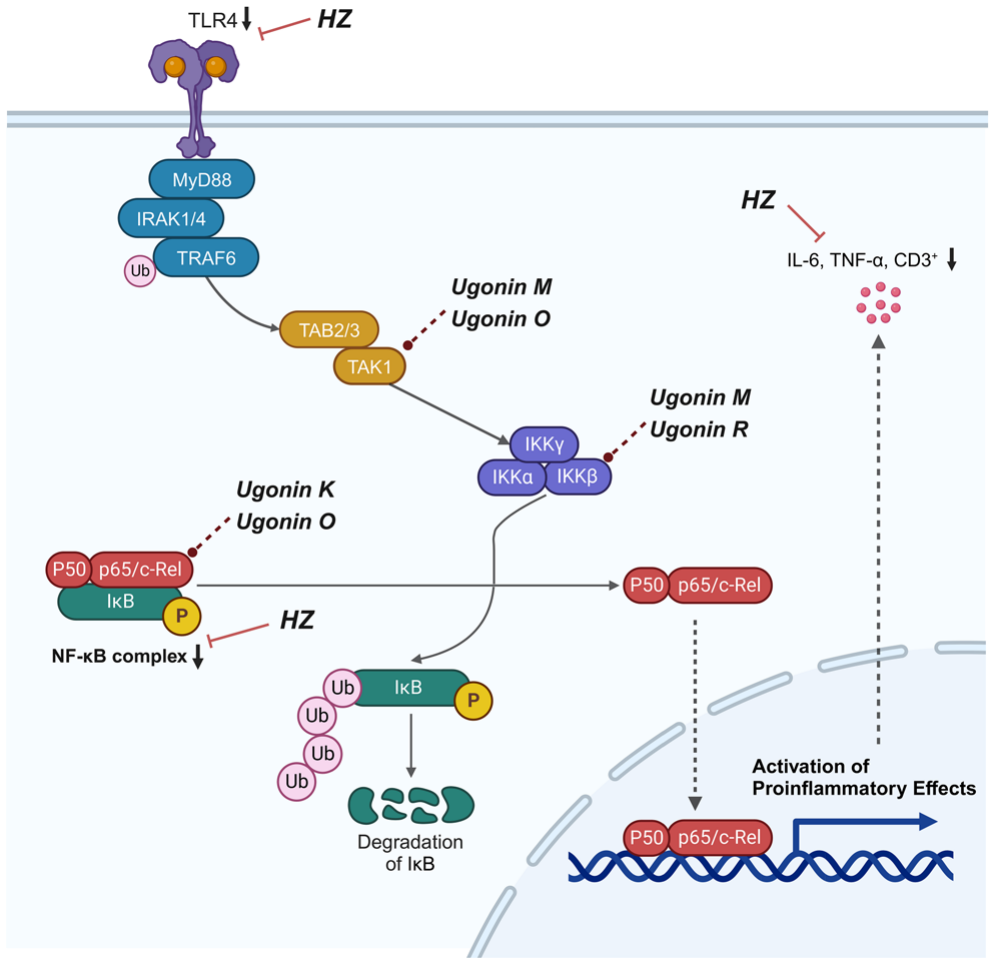
The mechanism of HZ in UC involves the TLR4/NF‐κB pathway. The anti‐inflammatory properties of HZ on UC are mediated through the TLR4/NF‐κB signaling pathway, encompassing the inhibition of cytokine production and prevention of T cell activation and differentiation. HZ achieves a downregulation of TLR4 and NF‐κB, resulting in reduced cellular release of TNF and IL‐6. Subsequent molecular docking simulation analysis reveals that the pure compounds of HZ, namely UgoninM, O, R, and K, exert their inhibitory effects on the TLR4/NF‐κB signaling pathway by influencing proteins such as TAK1, IKKβ, and RELA. Consequently, this impedes the degradation of the NF‐κB trimer, preventing its entry into the cell nucleus and activating pro‐inflammatory effects. On another note, CD3+ T cells, when associated, play a role in enhancing the production of specific antibodies by immune cells through the secretion of cytokines such as IL‐6 and TNF, thereby amplifying the extent of humoral immune responses.

We performed a comparative analysis of the Toll‐like receptor signaling pathway and the NF‐κB signaling pathway using the DAVID database. Subsequently, we identified proteins associated with shared pathways and validated the interactions of eight proteins and their interaction characteristics using IPA software (Figure S4). All 18 compounds of HZ listed in Table S3 were selected as ligands and subjected to docking studies with critical proteins, including TLR4, RELA, NFKBIA, MyD88, IRAK4, TRAF4, MAP3K7, and IKBKB, which are presumed to play crucial roles in the TLR4/NF‐κB pathway. The absolute value of the binding energy reflects the affinity between the compounds and proteins, with higher absolute values indicating stronger affinity. In Figure S5, we defined receptor–ligand interactions with binding affinities greater than the absolute value of 9.8 kcal/mol as high‐intensity combinations (Lin et al. 2024). Accordingly, Ugonin M—IKBKB (−11.1 kcal/mol), Ugonin M—MAP3K7 (−10.5 kcal/mol), Ugonin O—MAP3K7 (−10.1 kcal/mol), Ugonin O—RELA (−10.4 kcal/mol), Ugonin R—IKBKB (−9.8 kcal/mol), and Ugonin K—RELA (−9.8 kcal/mol) were identified as strong binding pairs. These interactions were further supported by the visualization of polar hydrogen bonds, indicating stable conformations within the protein–ligand complexes. Illustrated in Figure 7A–F are the molecular docking results depicting specific hydrogen bond interactions between the target protein MAP3K7 and the compounds Ugonin M (ASP‐329, ASP‐272) and Ugonin O (GLY‐350, GLU‐225). Additionally, β‐sitosterol is shown to form hydrogen bonds with the amino acid residues of Ugonin M (HIS‐409) and Ugonin R (LYS‐438). Furthermore, RELA is observed to form hydrogen bonds with the amino acid residues of Ugonin R (ALA‐107, SER‐111, GLU‐105) and Ugonin O (ALA‐107). These interactions highlight the intricate molecular relationships between the active compounds and their respective protein targets. The computer‐simulated results of molecular docking complement the network pharmacology analysis derived from both in vivo and in vitro studies, elucidating how the pure compounds in HZ influence UC and laying the groundwork for further exploration of its pharmacological mechanisms. The comprehensive results of the integrated studies on the mechanism of HZ in treating UC, particularly involving the TLR4/NF‐κB pathway, are visually presented in Figure 8, synthesizing the results from the IPA pathway analysis and affinity comparison.

## 4 Discussion

This study was motivated by the need to understand UC's immunopathogenesis better and explore the potential of natural compounds as alternative therapeutic options. UC, a chronic disease marked by relapsing inflammation, leads to progressive intestinal damage due to dysregulated immune responses, which activate leukocytes and epithelial cells, driving cytokine overproduction and oxidative stress (Biasi et al. 2011; Hoensch and Weigmann 2018). Flavonoids and plant sterol compounds exhibit anti‐inflammatory potential by activating regulatory pathways in response to oxidative and inflammatory stimuli, with NF‐κB acting as a central downstream effector (López‐García et al. 2019; Cheon et al. 2006). These compounds engage Toll‐like receptors on immune cell surfaces, translocate into the nucleus, bind to aryl hydrocarbon receptors, and activate aryl hydrocarbon response elements, ultimately promoting the production of protective enzymes and cytokines within the anti‐inflammatory system (Furumatsu et al. 2011).

Through literature integration, we identified 18 critical phytochemicals in HZ extract, including flavonoids (quercetin derivatives, ugonin, ugonstilbene), fatty acids (eicosapentaenoic acid, tetracosanoic acid), plant sterols (β‐sitosterol, stigmasterol), and phospholipids. PPI analysis revealed that these compounds effectively modulate inflammatory pathways, particularly IL‐6 and TNF, with significant reductions observed in DSS‐induced colitis models. HZ inhibits IL‐6 and TNF‐α signaling by regulating the conversion of arachidonic acid to PGE2, deactivating cPLA2, and suppressing NF‐κB binding, which subsequently downregulates COX‐2 expression, thereby reducing inflammation (Wu et al. 2018; Tsai et al. 2021; Echizen et al. 2016).

KEGG pathway analysis and in vitro/in vivo experiments identified the Toll‐like receptor and NF‐κB pathways, with TLR4 and NF‐κB as key markers mediating HZ's therapeutic effects on UC. In a DSS‐induced colitis model, HZ dose‐dependently reduced the upregulation of TLR4 and NF‐κB, alleviating inflammation. Additionally, findings from an observational case–control study in Chinese populations revealed a significant positive correlation between the severity of colonic mucosal ulceration, the DAI score, and the upregulation of TLR4 and RELA (Yu et al. 2011). UC patients exhibited increased TLR4 immunoreactivity in mucosal epithelial cells compared to healthy controls, and TLR4 activates NF‐κB, which triggers phosphorylation and degradation of inhibitory proteins, upregulating proinflammatory cytokines and chemokines, thereby sustaining NF‐κB activity and exacerbating inflammation (Fan and Liu 2015; Andersen et al. 2011). Mechanistically, TLR4 signals through adaptor proteins (MyD88, IRAK4, TRAF6, MAP3K7) to activate NF‐κB. This process includes IκB degradation, NF‐κB nuclear translocation, and increased expression of inflammatory genes such as COX‐2, TNF‐α, IL‐1β, IL‐6, and IL‐8 (de Oliveira et al. 2021; Cui et al. 2014; DiPerna et al. 2004).

Molecular docking analysis in our study confirmed that Ugonin M, O, K, and R—essential compounds in HZ—effectively target critical proteins within Toll‐like receptor cascades, specifically TAK1 (MAP3K7), IKKβ, and RELA, as demonstrated in Figure 8. These cascades regulate inflammatory responses primarily through pathways like MAPK and NF‐κB (Kumar et al. 2009). TAK1 serves as a pivotal upstream regulator in these pathways. It phosphorylates and activates IKKβ, which marks IκB for degradation via ubiquitination. This process releases the NF‐κB p50‐RelA complex, enabling its nuclear translocation to activate proinflammatory genes like TNF‐α, IL‐6, and COX‐2, thereby amplifying inflammation (Gilmore 1999; Gerondakis et al. 1999). Experimental data further demonstrated that HZ water extract, containing Ugonin K, reduced the phosphorylation of NF‐κB subunits (IκB‐α and p65) and mitigated MAPK pathway activation in a lipopolysaccharide‐induced lung injury model (Liou et al. 2017). These findings highlight HZ's potential to modulate inflammation through multiple molecular pathways.

Studies show that water‐extracted HZ reduces T cell activation and inflammation (Zhou et al. 2012). In the DSS‐induced UC model, inhibiting the TLR4‐related pathway decreases activated macrophages, neutrophil‐related inflammatory mediators, and T‐cell activation levels, emphasizing HZ's role in immune modulation. In the DSS‐induced UC model, inhibiting the TLR4‐related pathway effectively reduces activated macrophages, neutrophil‐related inflammatory mediators and T‐cell activation levels (Furumatsu et al. 2011). CD3, a key component in T cell immune responses, also forms part of the antigen recognition and signal transduction system necessary for T cell activation and differentiation. Blockers such as anti‐CD3 monoclonal antibodies have shown clinical benefits in severe UC patients unresponsive to corticosteroids (Vossenkämper et al. 2014; Kuhn and Weiner 2016). Our immunofluorescent staining experiments confirm that HZ significantly reduces CD3 expression in UC colon mucosal tissues and decreases IL‐6 release. While the specificity of HZ may not yet match that of monoclonal antibodies like Visilizumab, it offers a promising alternative therapy for UC patients who do not respond to conventional treatments (Plevy et al. 2007).

However, our study has several limitations due to budget and time constraints. First, we did not include a positive control group in our in vivo experiments, which is essential for comparing the efficacy of HZ as an alternative treatment. To address this, future studies on HZ's pure compounds should incorporate established immunomodulators, such as 5‐aminosalicylic acid, Azathioprine, or Cyclosporine, to provide a more robust comparison of effectiveness (Chen, Zhang, et al. 2019). Second, although core compounds were identified via molecular docking, their anti‐inflammatory and anti‐ulcer effects were not experimentally validated. Future studies will assess intestinal barrier integrity by measuring tight junction proteins (e.g., ZO‐1, occludin) and intestinal permeability (e.g., FITC‐dextran) to evaluate if HZ and its components improve barrier function in UC mice (Wang et al. 2023). Third, the small sample size per group (*n* = 5) may have limited the statistical power and generalizability of our findings, thereby increasing the risk of Type II error. Moreover, because the DSS‐induced colitis model is characterized by high mortality and considerable individual variability, these factors may have further compromised the robustness of the results when combined with a small group size. To address this limitation, future studies will prioritize recruiting sufficiently large cohorts to enhance statistical power. If larger sample sizes are not feasible, we will instead employ non‐parametric approaches such as the Kruskal–Wallis test or permutation tests to reduce distributional assumptions and improve the reliability of the results.

## 5 Conclusion

In summary, our study provides novel insights into the anti‐inflammatory effects of HZ. We confirmed that HZ exerts therapeutic effects via the TLR4/NF‐κB pathway in a UC mouse model, reducing vital inflammatory markers, including IL‐6, TNF, and CD3 expression. Importantly, we observed that HZ's anti‐cell proliferative effects are not associated with apoptosis, marking a distinct mechanism compared to other anti‐inflammatory agents. Furthermore, we identified a significant role for ugonin compounds in mediating these effects, particularly in the gastrointestinal tract, as demonstrated by binding solid affinity results. These findings contribute to a deeper understanding of the molecular mechanisms through which HZ may alleviate UC, highlighting its potential as a therapeutic agent.

## Author Contributions

**Chih‐Ting Lin:** conceptualization (supporting), data curation (supporting), methodology (lead), resources (supporting), software (lead), visualization (lead), writing – original draft (lead). **Lung‐Yuan Wu:** conceptualization (lead), investigation (supporting), project administration (supporting), resources (supporting), supervision (lead), writing – review and editing (supporting). **Li‐Wei Lin:** data curation (supporting), formal analysis (supporting), funding acquisition (supporting), methodology (lead), resources (lead), software (supporting). **Li‐Ching Chang:** conceptualization (supporting), data curation (supporting), formal analysis (lead), resources (lead), validation (lead). **Fan‐Shiu Tsai:** conceptualization (supporting), investigation (lead), methodology (lead), project administration (lead), validation (lead), visualization (lead), writing – review and editing (lead).

## Ethics Statement

This study adheres strictly to ethical guidelines, particularly the 3R principles (Replacement, Reduction, and Refinement) for the humane use of animals in research. All in‐life rat studies were conducted in facilities approved by the Association for Assessment and Accreditation of Laboratory Animal Care and were further approved by the I‐Shou University Institutional Animal Care and Use Committee (Approval numbers IACUC‐ISU‐102025). Furthermore, we affirm that the experiments involving our experimental animals have been conducted and reported in accordance with the ARRIVE guidelines (https://arriveguidelines.org). For the euthanasia of mice, carbon dioxide (CO_2_) asphyxiation was employed in a designated sacrificial cage, where the CO_2_ flow rate was set at 3.4 (40% of the box volume per minute), and the animals were confirmed dead after approximately 5–6 min. This method aligns with ethical standards and guidelines of 3R principles to minimize animal discomfort and distress during experimentation. All plant experiments and protocols followed the relevant institutional, national, and international guidelines and legislation.

## Consent

The authors have nothing to report.

## Conflicts of Interest

The authors declare no conflicts of interest.

## Supporting information


**Figure S1:** fsn371139‐sup‐0001‐FigureS1.jpg.

**Figure S2:** fsn371139‐sup‐0002‐FigureS2.jpg.

**Figure S3:** fsn371139‐sup‐0003‐FigureS3.jpg.

**Figure S4:** fsn371139‐sup‐0004‐FigureS4.jpg.

**Figure S5:** fsn371139‐sup‐0005‐FigureS5.jpg.

**Figure S6:** fsn371139‐sup‐0006‐FigureS6.jpg.

**Table S1:** fsn371139‐sup‐0007‐TableS1‐S7.docx.

## Data Availability

All data generated or analyzed during this study is included in this article and its Supporting Information files, ensuring full availability for transparency and reproducibility.
