# Supplementary figures and images for: Combining Network Pharmacology, Molecular Docking, and Integrative Studies to Explore the Mechanism of Helminthostachys zeylanica in Alleviating Ulcerative Colitis
Source: Food Sci Nutr. 2025 Oct 28;13(11):e71139. doi: 10.1002/fsn3.71139 (PMC12560106; doi:10.1002/fsn3.71139)

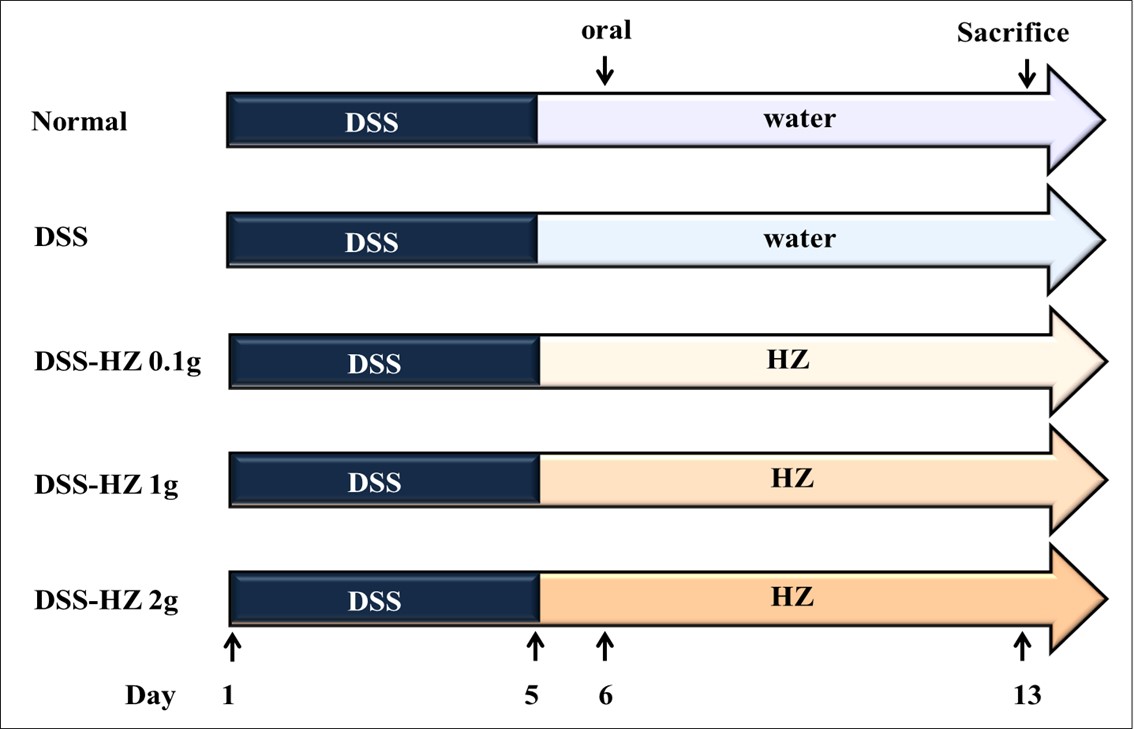

Supplement: Supplementary file 1 — Figure S1: fsn371139‐sup‐0001‐FigureS1.jpg. [file FSN3-13-e71139-s003.jpg]

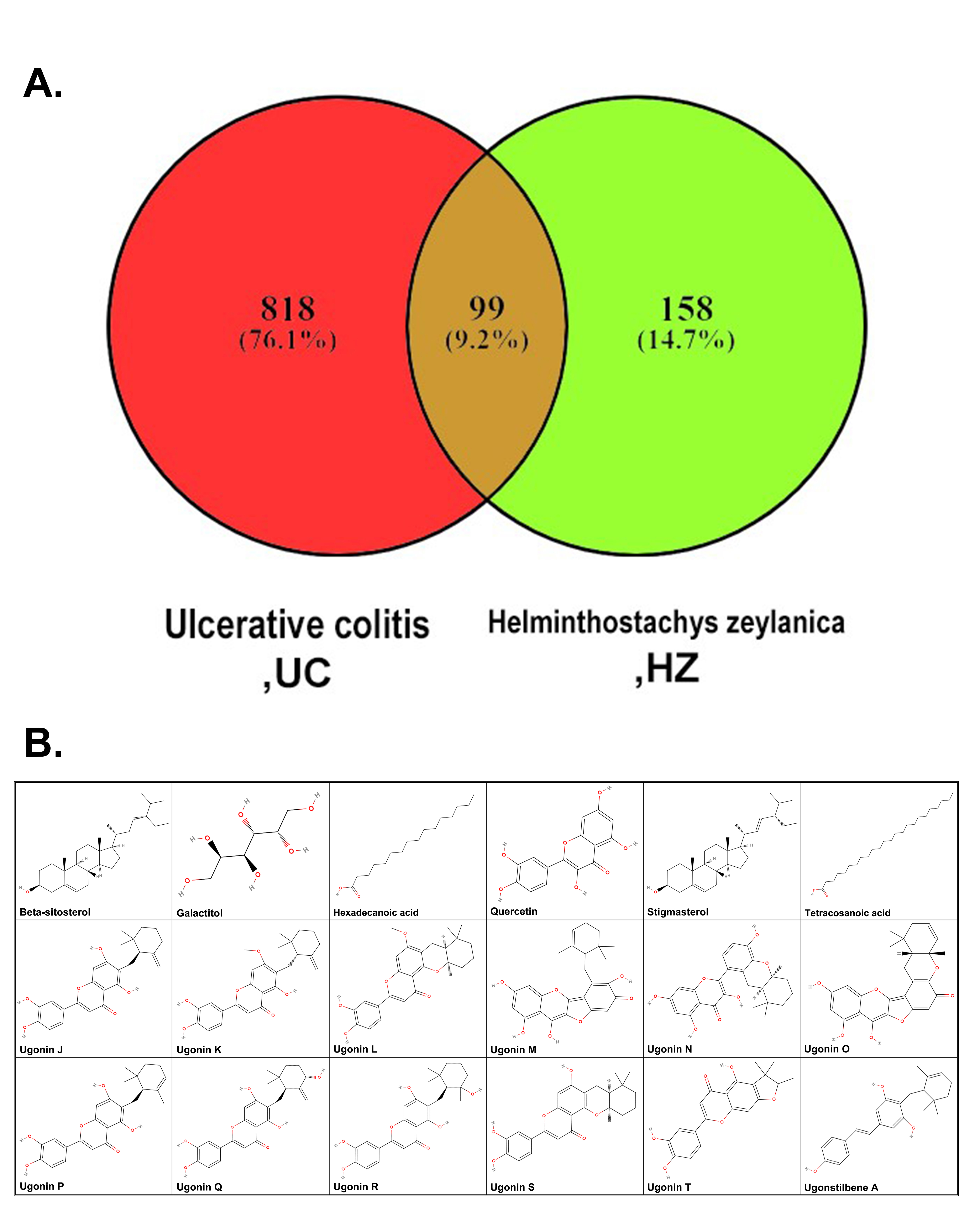

Supplement: Supplementary file 2 — Figure S2: fsn371139‐sup‐0002‐FigureS2.jpg. [file FSN3-13-e71139-s002.jpg]

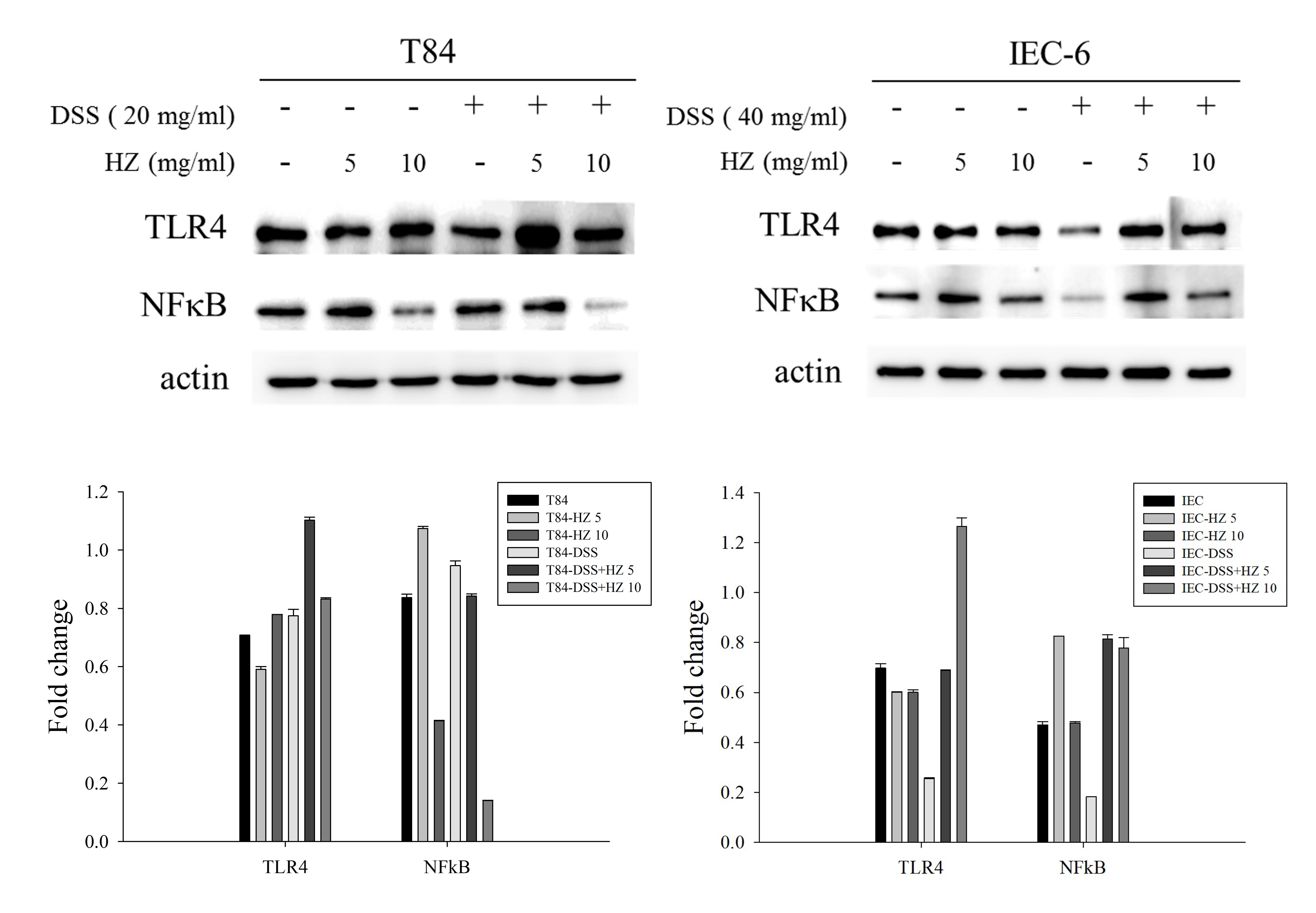

Supplement: Supplementary file 3 — Figure S3: fsn371139‐sup‐0003‐FigureS3.jpg. [file FSN3-13-e71139-s006.jpg]

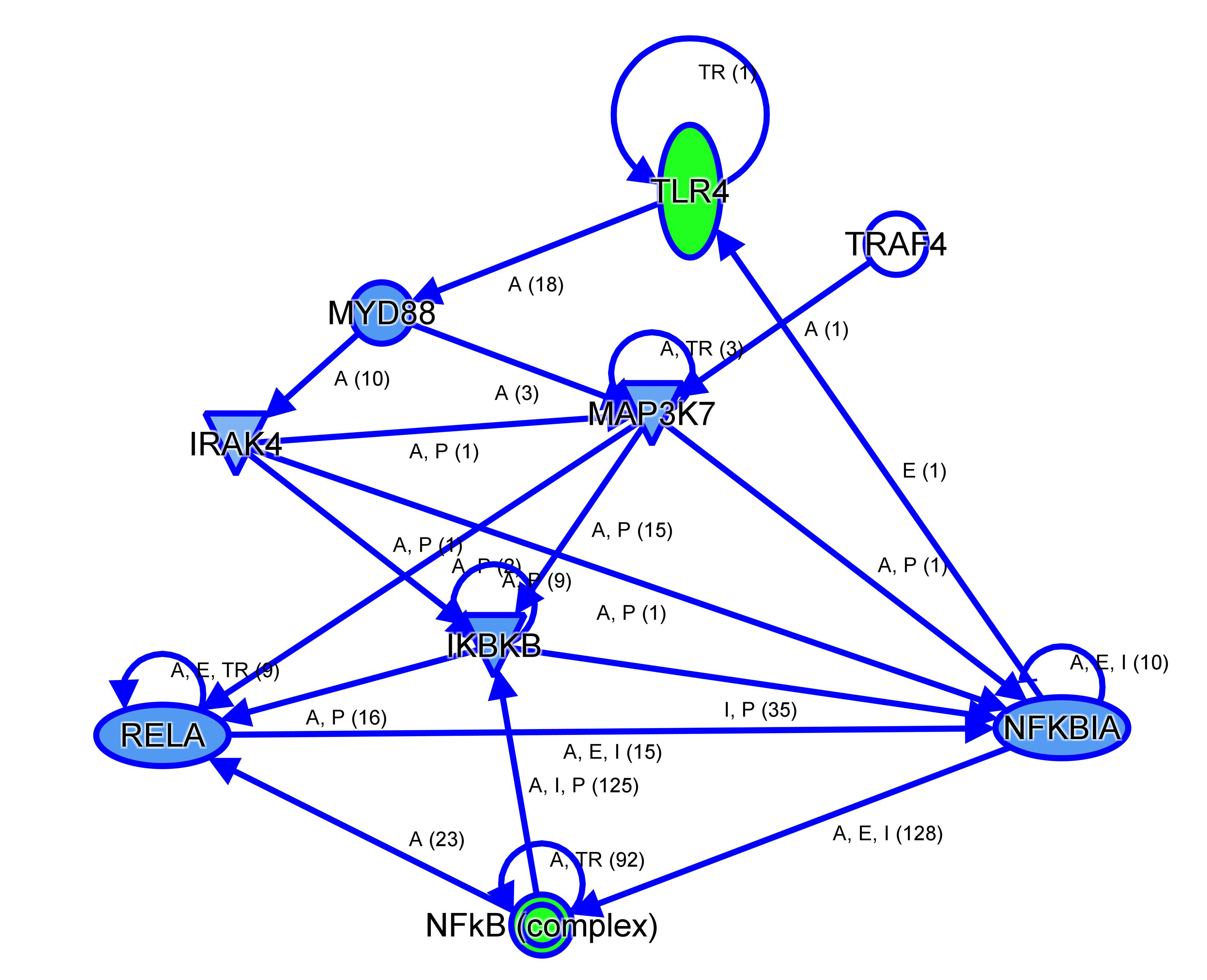

Supplement: Supplementary file 4 — Figure S4: fsn371139‐sup‐0004‐FigureS4.jpg. [file FSN3-13-e71139-s005.jpg]

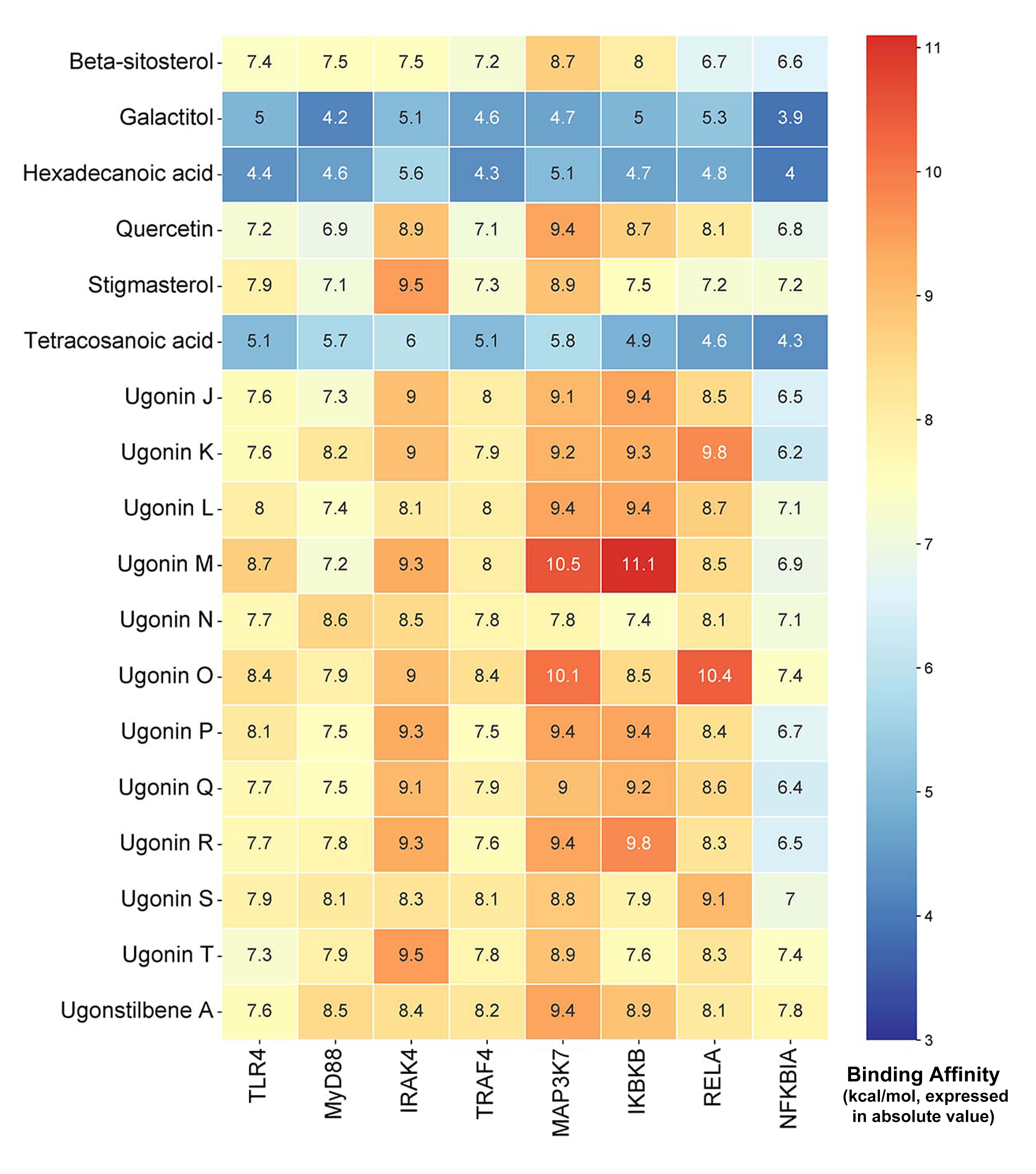

Supplement: Supplementary file 5 — Figure S5: fsn371139‐sup‐0005‐FigureS5.jpg. [file FSN3-13-e71139-s007.jpg]

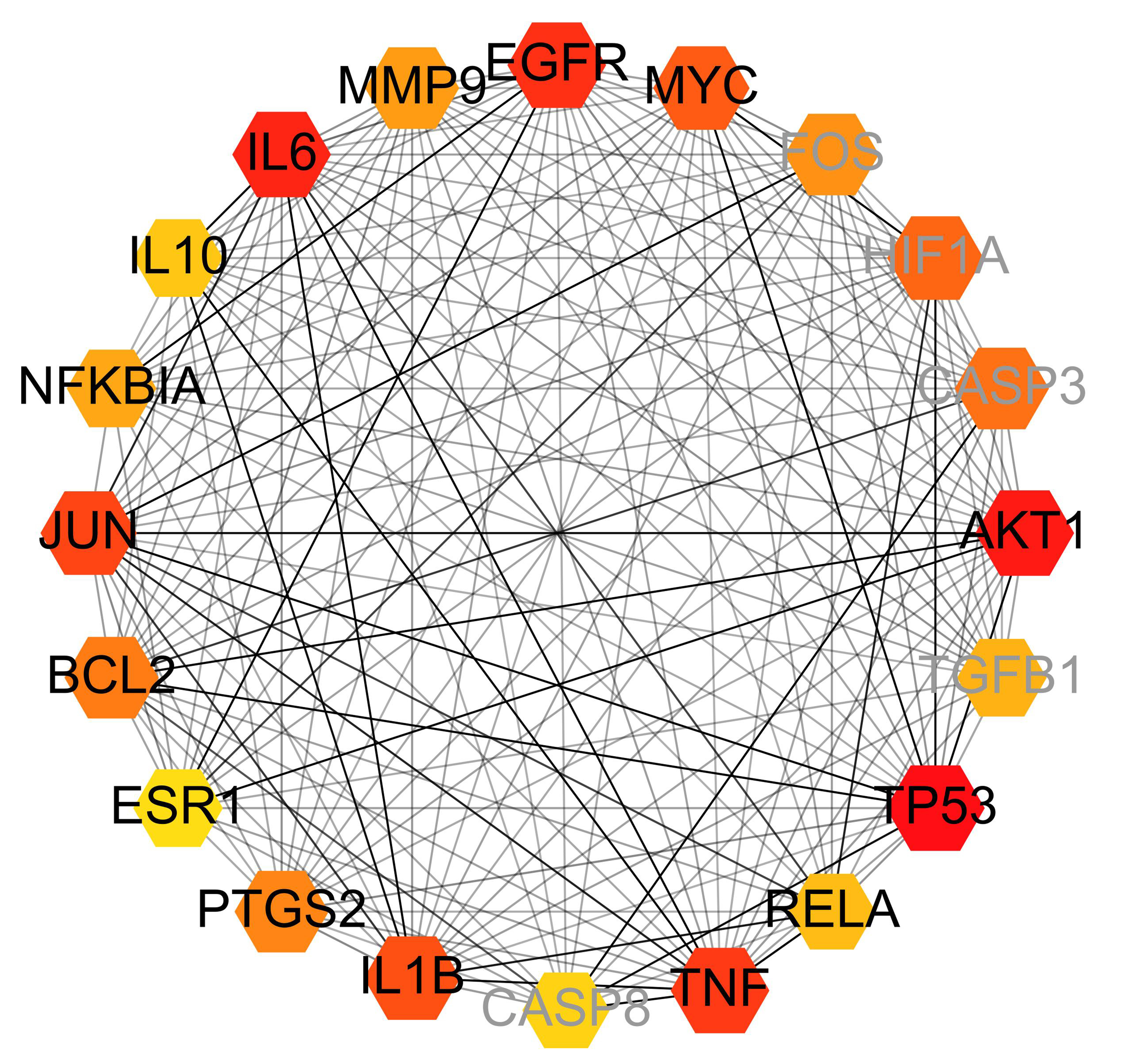

Supplement: Supplementary file 6 — Figure S6: fsn371139‐sup‐0006‐FigureS6.jpg. [file FSN3-13-e71139-s001.jpg]
