# Supplementary material for: Combining Network Pharmacology, Molecular Docking, and Integrative Studies to Explore the Mechanism of Helminthostachys zeylanica in Alleviating Ulcerative Colitis
Source: Food Sci Nutr. 2025 Oct 28;13(11):e71139. doi: 10.1002/fsn3.71139 (PMC12560106; doi:10.1002/fsn3.71139)
Supplement: Supplementary file 7 — Table S1: fsn371139‐sup‐0007‐TableS1‐S7.docx. [file FSN3-13-e71139-s004.docx]

**Supplementary Tables**

| **SAMPLE** | **PDBID** | **Method** | **Organism(s)** | **Resolution** | **R-ValueFree** |
| --- | --- | --- | --- | --- | --- |
| TLR4 | 4G8A | X-ray Diffraction | Homo sapiens | 2.40Å | 0.249 |
| RELA | 1IKN | X-ray Diffraction | Homo sapiens | 2.30Å | 0.227 |
| NFKBIA | 1NFI | X-ray Diffraction | Homo sapiens | 2.70Å | 0.265 |
| MyD88 | 4DOM | X-ray Diffraction | Homo sapiens | 1.80 Å | 0.216 |
| IRAK4 | 6O8U | X-ray Diffraction | Homo sapiens | 1.80 Å | 0.255 |
| TRAF4 | 3ZJB | X-ray Diffraction | Homo sapiens | 1.84 Å | 0.2 |
| MAP3K7 | 5JGA | X-ray Diffraction | Homo sapiens | 2.00Å | 0.221 |
| IKBKB | 4KIK | X-ray Diffraction | Homo sapiens | 2.83Å | 0.236 |

**Table. S1. The pre-docking preparation parameters of target proteins**

| No. |  | Compound | References | Extraction methods | Measure tools |
| --- | --- | --- | --- | --- | --- |
| HZ1 |  | Beta-sitosterol | Huang, Y.-C., Hwang, T.-L., Yang, Y.-L., Wu, S.-H., Hsu, M.-H., Wang, J.-P., Chen, S.-C., Huang, L.-J., & Liaw, C.-C. (2010). Acetogenin and prenylated flavonoids from Helminthostachys zeylanica with inhibitory activity on superoxide generation and elastase release by neutrophils. Planta Medica, 76(5), 447–453. https://doi.org/10.1055/s-0029-1186221 | MeOH | HPLC |
| HZ2 |  | Galactitol | Shah, A. B., Liu, Y.-L., & Kuo, Y.-H. (2020). Effectiveness of cyclohexyl functionality in ugonins from Helminthostachys zeylanica to PTP1B and α-glucosidase inhibitions. International Journal of Biological Macromolecules, 165(Pt B), 1822–1831. https://doi.org/10.1016/j.ijbiomac.2020.10.061 | Unknow | Unknow |
| HZ3 |  | Palmitic Acid | Huang, Y.-C., Hwang, T.-L., Yang, Y.-L., Wu, S.-H., Hsu, M.-H., Wang, J.-P., Chen, S.-C., Huang, L.-J., & Liaw, C.-C. (2010). Acetogenin and prenylated flavonoids from Helminthostachys zeylanica with inhibitory activity on superoxide generation and elastase release by neutrophils. Planta Medica, 76(5), 447–453. https://doi.org/10.1055/s-0029-1186221 | MeOH | HPLC |
| HZ4 |  | Quercetin | Wu, K.-C., Kao, C.-P., Ho, Y.-L., & Chang, Y.-S. (2017). Quality control of the root and rhizome of Helminthostachys zeylanica (Daodi-Ugon) by HPLC using quercetin and ugonins as markers. Molecules, 22(7), 1115. https://doi.org/10.3390/molecules22071115 | Ethanol | HPLC |
| HZ5 |  | Stigmasterol | Huang, Y.-C., Hwang, T.-L., Yang, Y.-L., Wu, S.-H., Hsu, M.-H., Wang, J.-P., Chen, S.-C., Huang, L.-J., & Liaw, C.-C. (2010). Acetogenin and prenylated flavonoids from Helminthostachys zeylanica with inhibitory activity on superoxide generation and elastase release by neutrophils. Planta Medica, 76(5), 447–453. https://doi.org/10.1055/s-0029-1186221 | MeOH | HPLC |
| HZ6 |  | Stearic acid | Huang, Y.-C., Hwang, T.-L., Yang, Y.-L., Wu, S.-H., Hsu, M.-H., Wang, J.-P., Chen, S.-C., Huang, L.-J., & Liaw, C.-C. (2010). Acetogenin and prenylated flavonoids from Helminthostachys zeylanica with inhibitory activity on superoxide generation and elastase release by neutrophils. Planta Medica, 76(5), 447–453. https://doi.org/10.1055/s-0029-1186221 | MeOH | HPLC |
| HZ7 |  | Ugonin J | Liou, C.-J., Huang, Y.-L., Huang, W.-C., Yeh, K.-W., Huang, T.-Y., & Lin, C.-F. (2017). Water extract of Helminthostachys zeylanica attenuates LPS-induced acute lung injury in mice by modulating NF-κB and MAPK pathways. Journal of Ethnopharmacology, 199, 30–38. https://doi.org/10.1016/j.jep.2016.03.057 | Water | HPLC |
| HZ8 |  | Ugonin K | Chan, L. P., Chou, T. H., Wang, G. H., Tseng, Y. P., Chen, P. J., Cheng, D. L., & Liang, C. H. (2013). Ugonin K induces cell cycle arrest and apoptosis through reactive oxygen species/apoptosis signal pathway in human skin cancer cells. Advanced Materials Research, 690–693, 1422–1425. | Ethanol | HPLC |
| HZ9 |  | Ugonin L | Liu, C.-L., Ho, T.-L., Fang, S.-Y., Guo, J.-H., Wu, C.-Y., Fong, Y.-C., Liaw, C.-C., & Tang, C.-H. (2023). Ugonin L inhibits osteoclast formation and promotes osteoclast apoptosis by inhibiting the MAPK and NF-κB pathways. Biomedicine & Pharmacotherapy, 166, 115392. https://doi.org/10.1016/j.biopha.2023.115392 | MeOH | Column Chromatography |
| HZ10 |  | Ugonin M | Wu, K.-C., Huang, S.-S., Kuo, Y.-H., Ho, Y.-L., Yang, C.-S., Chang, Y.-S., & Huang, G.-J. (2017). Ugonin M, a Helminthostachys zeylanica constituent, prevents LPS-induced acute lung injury through TLR4-mediated MAPK and NF-κB signaling pathways. Molecules, 22(4), 573. https://doi.org/10.3390/molecules22040573 | Ethanol | HPLC |
| HZ11 |  | Ugonin N | Hsu, Y.-L., Liu, Y.-C., & Yang, Y.-L. (2016). Anti-inflammatory flavonoids from the rhizomes of Helminthostachys zeylanica. Journal of Natural Products, 79(1), 1–7. https://doi.org/10.1021/np500778v | Water | HPLC |
| HZ12 |  | Ugonin O | Hsu, Y.-L., Liu, Y.-C., & Yang, Y.-L. (2017). Anti-inflammatory and antiosteoporosis flavonoids from the rhizomes of Helminthostachys zeylanica. Journal of Natural Products, 80(2), 246–253. https://doi.org/10.1021/acs.jnatprod.6b00956 | Water | MPLC/HPLC |
| HZ13 |  | Ugonin P | Hsu, Y.-L., Liu, Y.-C., & Yang, Y.-L. (2016). Anti-inflammatory flavonoids from the rhizomes of Helminthostachys zeylanica. Journal of Natural Products, 79(1), 1–7. https://doi.org/10.1021/np500778v | Water | HPLC |
| HZ14 |  | Ugonin Q | Hsu, Y.-L., Liu, Y.-C., & Yang, Y.-L. (2017). Anti-inflammatory and antiosteoporosis flavonoids from the rhizomes of Helminthostachys zeylanica. Journal of Natural Products, 80(2), 246–253. https://doi.org/10.1021/acs.jnatprod.6b00956 | Water | MPLC/HPLC |
| HZ15 |  | Ugonin R | Hsu, Y.-L., Liu, Y.-C., & Yang, Y.-L. (2017). Anti-inflammatory and antiosteoporosis flavonoids from the rhizomes of Helminthostachys zeylanica. Journal of Natural Products, 80(2), 246–253. https://doi.org/10.1021/acs.jnatprod.6b00956 | Water | MPLC/HPLC |
| HZ16 |  | Ugonin S | Shah, A. B., Liu, Y.-L., & Kuo, Y.-H. (2020). Effectiveness of cyclohexyl functionality in ugonins from Helminthostachys zeylanica to PTP1B and α-glucosidase inhibitions. International Journal of Biological Macromolecules, 165(Pt B), 1822–1831. https://doi.org/10.1016/j.ijbiomac.2020.10.061 | MeOH | Column Chromatography |
| HZ17 |  | Ugonin T | Hsu, Y.-L., Liu, Y.-C., & Yang, Y.-L. (2017). Anti-inflammatory and antiosteoporosis flavonoids from the rhizomes of Helminthostachys zeylanica. Journal of Natural Products, 80(2), 246–253. https://doi.org/10.1021/acs.jnatprod.6b00956 | Water | MPLC/HPLC |
| HZ18 |  | Ugonstilbene A | Liu, Y.-L., & Hsu, Y.-L. (2022). Total syntheses, absolute configurations, and cytotoxicity evaluation of ugonstilbenes A, B, and C from the rhizomes of Helminthostachys zeylanica. Journal of Natural Products, 85(6), 1702–1710. https://doi.org/10.1021/acs.jnatprod.2c00919 | Ethanol | Column Chromatography |

**Table. S2.** Active Compounds of Helminthostachys zeylanica (HZ) with Corresponding Extraction Methods and Mass Spectrometry Identification

| Compound Name | No. | Related Target Gene |
| --- | --- | --- |
| Beta-sitosterol | HZ1 | NF, TGFB1, PTGS2, PTGS1, BAX, BCL2, PIK3CG, CASP3,CASP8, PON1,JUN, SLC6A4, AKT1, MAP2K1 |
| Galactitol | HZ2 | IL6, PTPN2, PTGER4, AKT1, FOSB |
| Palmitic Acid | HZ3 | TNF, IL6, IL10, SLC22A5, PTGS2, PTGS1, PTEN, BCL2, CTSD |
| Quercetin | HZ4 | TNF, IL6, IL10, IL1Β, MPO, IFNG, TGFB1, IL2, CRP, TP53, PTGS2, AKT1, PPARG,COL18A1, RELA, PTGS1, CHEK2, MMP1, MMP9, ICAM1, EGF, CDKN2A, F2, DUOX2,CRYGC, CD40LG, MAP3K7, NFE2L2, IL1A, EGFR, F3,STAT1, MMP3, CDKN1A, CXCL10, CCND1, BAX, MMP2, VCAM1, SPP1, ERBB2, CYP3A4, SOD1, PTEN, SELE, SERPINE1, HMOX1, BCL2, GSTP1, CLDN4, XDH, CXCL2, NCF1, GSTM1, NR1I2, IGF2, HIF1A, ALOX5, ODC1, NFKBIA, PIK3CG, MYC, PLAU, BIRC5, CASP3, ABCG2, NOS3, THBD, IRF1, CASP8, PON1, JUN, BCL2L1, PARP1, FOS, NQO1, CYP1A2, AHR, CYP1A1, PTGER3, MAP2K1, CTSD, AKT1 |
| Stigmasterol | HZ5 | PTGS2, PTGS1, PLAU, FOSB |
| Stearic acid | HZ6 | DUOX2, NCF1 |
| Ugonin J | HZ7 | IL6, ADA, PTGER4, ESR1, ESR2, MAP2K1 |
| Ugonin K | HZ8 | TNF, IL6, AKT1, ADA, PTGER4, NQO1 |
| Ugonin L | HZ9 | TNF, IL6, NLRP3, BRAF, ADA, PTGER4, MAP2K1 |
| Ugonin M | HZ10 | TNF, IL6, NLRP3, GPR35, ADA, PTGER4, NQO1 |
| Ugonin N | HZ11 | TNF, IL6, ADA, PTGER4, SRC, FOSB |
| Ugonin O | HZ12 | IL6, ADA, PTGER4, SRC |
| Ugonin P | HZ13 | IL6, ADA, PTGER4, SRC, HTR3A, PDE4A |
| Ugonin Q | HZ14 | TNF, IL6, GPR35, PTGER4, MALT1 |
| Ugonin R | HZ15 | TNF, IL6, NLRP3, GPR35, ADA, PTGER4, MMP13, MALT1 |
| Ugonin S | HZ16 | TNF, IL6, BRAF, ADA, PTGER4, MAP2K1 |
| Ugonin T | HZ17 | TNF, IL6, ADA, PTGER4, CYP1A2, MAP2K1 |
| Ugonstilbene A | HZ18 | IL6, ADA, PTGER4, ESR1, ESR2 |

**Table. S3.** The active compounds of HZ and their corresponding potential target gene

| Compound Name | No. | Degree | Betweenness Centrality | Clossness Centrality |
| --- | --- | --- | --- | --- |
| Quercetin | HZ4 | 83 | 0.868 | 0.694 |
| Beta-sitosterol | HZ1 | 14 | 0.045 | 0.35 |
| Palmitic Acid | HZ3 | 9 | 0.031 | 0.368 |
| Ugonin R | HZ15 | 8 | 0.037 | 0.356 |
| Ugonin L | HZ9 | 7 | 0.019 | 0.361 |
| Ugonin M | HZ10 | 7 | 0.017 | 0.354 |
| Ugonin P | HZ13 | 6 | 0.039 | 0.352 |
| Ugonin J | HZ7 | 6 | 0.024 | 0.359 |
| Ugonin N | HZ11 | 6 | 0.017 | 0.354 |
| Ugonin S | HZ16 | 6 | 0.012 | 0.359 |
| Ugonin T | HZ17 | 6 | 0.008 | 0.359 |
| Ugonin K | HZ8 | 6 | 0.007 | 0.359 |
| Galactitol | HZ2 | 5 | 0.024 | 0.359 |
| Ugonin Q | HZ14 | 5 | 0.013 | 0.35 |
| Ugonstilbene A | HZ18 | 5 | 0.013 | 0.35 |
| Stigmasterol | HZ5 | 4 | 0.006 | 0.317 |
| Ugonin O | HZ12 | 4 | 0.005 | 0.348 |
| Stearic acid | HZ6 | 2 | 0.004 | 0.295 |

**Table. S4.** Topological analysis of the core active compounds in components-compounds-targets network. (Sorted on Degree and Betweenness Centrality)

| Target Name | Degree | Betweenness Centrality | Clossness Centrality | Related KEGG Term |
| --- | --- | --- | --- | --- |
| IL6 | 53 | 0.104 | 0.679 | TNF signaling |
| TNF | 50 | 0.071 | 0.654 | TNF signaling |
| IL1Β | 48 | 0.061 | 0.645 | NF-κB signaling |
| JUN | 42 | 0.047 | 0.627 | TNF signaling |
| EGFR | 40 | 0.047 | 0.619 | None |
| RELA | 30 | 0.044 | 0.561 | NF-κB signaling |
| PTGS2 | 35 | 0.043 | 0.59 | NF-κB signaling |
| MMP9 | 39 | 0.042 | 0.614 | None |
| ESR1 | 28 | 0.039 | 0.568 | None |
| TP53 | 41 | 0.039 | 0.614 | None |
| NFKBIA | 25 | 0.037 | 0.565 | NF-κB signaling |
| MYC | 34 | 0.036 | 0.587 | None |
| AKT1 | 40 | 0.036 | 0.61 | Toll-like receptor signaling |
| BCL2 | 35 | 0.029 | 0.587 | NF-κB signaling |
| IL10 | 34 | 0.023 | 0.572 | T-cell receptor signaling |

**Table. S5.** Topological analysis of the core target protein in PPI network

| Category | Term | Fold Enrichment | Pvalue | Count |
| --- | --- | --- | --- | --- |
| BP | Negative regulation of Apoptotic process | 9.2 | 4.67E-16 | 24 |
| BP | Inflammatory response | 10.8 | 9.14E-17 | 23 |
| BP | Regulation of cell proliferation | 7.4 | 1.32E-11 | 20 |
| BP | Response to Lipopolysaccharide | 19.7 | 2.24E-13 | 14 |
| BP | Positive regulation of ERK1 and ERK2 cascade | 9.8 | 1.53E-07 | 11 |
| BP | Positive regulation of Cytokine production | 27.5 | 1.10E-09 | 9 |
| BP | Positive regulation of MAP kinase activity | 23.6 | 3.88E-09 | 9 |
| BP | Positive regulation of Protein kinase B signaling | 14.1 | 2.28E-07 | 9 |
| BP | positive regulation of NF-κB transcription factor activity | 9.8 | 1.66E-05 | 8 |
| CC | Cytoplasm | 1.6 | 1.64E-04 | 44 |
| CC | Nucleus | 1.5 | 6.63E-04 | 44 |
| CC | Plasma membrane | 1.6 | 1.81E-04 | 42 |
| CC | Extracellular exosome | 1.7 | 2.40E-02 | 18 |
| CC | Mitochondrion | 2.5 | 9.66E-04 | 17 |
| CC | Chromatin | 3 | 3.21E-04 | 15 |
| MF | Cytokine activity | 11.3 | 4.33E-08 | 11 |
| MF | Protein homodimerization activity | 4.8 | 1.30E-07 | 18 |
| MF | Transcription factor activity | 4.9 | 4.61E-06 | 14 |
| MF | Growth factor activity | 9.4 | 2.10E-05 | 8 |
| MF | Protein kinase activity | 5.2 | 1.18E-04 | 10 |
| MF | External side of plasma membrane | 4.5 | 3.55E-04 | 10 |
| MF | Zinc ion binding | 3.1 | 4.08E-04 | 14 |
| MF | Transcriptional activator activity | 4.1 | 6.52E-04 | 10 |
| MF | Calcium ion binding | 2.1 | 4.26E-02 | 8 |
| MF | Protein serine/threonine kinase activity | 2.9 | 4.99E-02 | 6 |
| MF | Endoplasmic reticulum membrane | 1.9 | 7.03E-02 | 10 |

**Table. S6.** Topological analysis of the GO functional enrichment analysis

| Term | Count | Pvalue | Fold Enrichment |
| --- | --- | --- | --- |
| TNF signaling pathway | 21 | 2.49E-19 | 16.7 |
| T cell receptor signaling pathway | 13 | 9.95E-13 | 11.3 |
| NF-κB signaling pathway | 15 | 4.16E-12 | 13.1 |
| Toll-like receptor signaling pathway | 14 | 6.76E-11 | 12.2 |
| HIF-1 signaling pathway | 15 | 8.04E-11 | 12.5 |
| MAPK signaling pathway | 19 | 2.84E-9 | 5.7 |
| PI3K-Akt signaling pathway | 20 | 5.53E-9 | 5.1 |
| JAK-STAT signaling pathway | 14 | 2.31E-8 | 7.6 |
| ErbB signaling pathway | 10 | 2.97E-7 | 10.7 |
| FoxO signaling pathway | 11 | 1.37E-6 | 7.6 |
| Chemokine signaling pathway | 11 | 4.09E-5 | 5.2 |
| B cell receptor signaling pathway | 7 | 2.87E-4 | 7.5 |
| Camp signaling pathway | 9 | 3.00E-03 | 3.6 |
| Ras signaling pathway | 8 | 1.41E-02 | 3 |
| Mtor signaling pathway | 5 | 2.96E-02 | 2.9 |

**Table. S7.** Topological analysis of pathways in KEGG enrichment analysis
